# Combining Carboxylic-Acid-Based Deep Eutectic Solvents and High Temperatures Enhances Phenolic Acid Extraction from Grape Pomace

**DOI:** 10.3390/antiox14060643

**Published:** 2025-05-27

**Authors:** Francesca Lorenzo, Marialaura Frisina, Sonia Bonacci, Monica Nardi, Manuela Oliverio, Antonio Procopio

**Affiliations:** Department of Health Sciences—A Green Food Research Center, University “Magna Græcia” of Catanzaro, Campus Universitario “S. Venuta”, Viale Europa, Loc. Germaneto, 88100 Catanzaro, Italy; francesca.lorenzo@studenti.unicz.it (F.L.); m.frisina@unicz.it (M.F.); s.bonacci@unicz.it (S.B.); monica.nardi@unicz.it (M.N.); procopio@unicz.it (A.P.)

**Keywords:** grape pomace, deep eutectic solvents, microwaves, phenolic acids

## Abstract

Phenolic acids are contained in grape pomace, mostly in a conjugate form, and can be a natural source of building blocks if they are efficiently hydrolyzed and extracted from the natural matrix. In this study, a comparative study based on the spectrophotometric evaluation of total phenolic content, hydroxycinnamic acid content, and anthocyanin content was performed on different carboxylic-acid-based NADES with different heating sources. Moreover, a quali–quantitative characterization of the bioactive molecules extracted was performed using UHPLC-ESI-HRMS. We found that the nature of the acidic component of the DES was crucial in selecting the family of molecules to be extracted; ChCl/oxalic acid 1:1 NADES, when combined with MAE at 100 °C, is the best medium for the in situ hydrolysis and extraction of phenolic acids from grape pomace. The ORAC test performed on natural extracts with and without NADES revealed a role for NADES components in antioxidant activity against the ROS of extracted bioactive phenols.

## 1. Introduction

**Figure 1:**
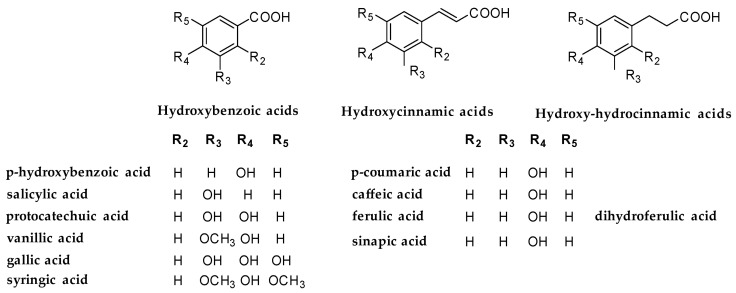
Chemical structure of the phenolic acids of grape pomace.

Phenolic acids are a ubiquitous class of bioactive molecules that occur in plants in both free and conjugate forms [1]. Their importance as vital dietary components in the human diet is well recognized by the scientific community, as well as their applicability in drug discovery as simple molecules protecting against oxidative stress, inflammation, and cancer [2,3]. Their activity is mainly due to the presence of a catechol group in a benzoic or a cinnamic acid (Figure 1) which is responsible for strong antioxidant activity.

We recently demonstrated that natural phenolic alcohols originate from the biosynthetic transformation of the amino acid tyrosine, namely hydroxytyrosol and tyrosol, and can be used as synthetic building blocks for a new generation of donepezil-like analogs that act as neuroprotective agents [4,5,6]. These results inspired the hypothesis that phenolic alcohols, originating from natural phenolic acids upon reduction, could provide new building blocks for the design of new compounds that are potentially active against neurodegeneration.

Grape pomace is an agricultural waste composed of grape skins, seeds, and stems, accumulated in tons during the processes of wine production [7]. The composition of grape pomace depends, among other factors, on the soil type, the grape variety, and agro-climatic conditions and is characterized by the presence of several classes of phenolic compounds, such as anthocyanins, flavonoids, tannins, and phenolic acids [7,8,9,10,11]. The latter are found as conjugated molecules that have been esterified with other hydroxy acids, mono/di-saccharides, and polymers, while a minor percentage are present as free monomers [1,7]. The overproduction of grape pomace is a problem for the wine production sector due to the high cost of its disposal. Conversely, the valorization of biomass from wineries can be a valuable solution from both ecological and economic perspectives. In the last 10 years, many extraction procedures have been proposed to recover bioactive molecules from grape pomace [7] exploiting classical solid–liquid extractions, including using hydroalcoholic solvents both alone [8,9,10] and in combination with alternative heating sources, such as microwaves (MAE) and ultrasound (UAE) [10,11,12,13], which allows for the enhancement of extraction yields, lowering the energy demands of the process. Most of them have been oriented toward the non-selective recovery of the most bioactive molecules, regardless of their specific chemical structure.

More recently, natural deep eutectic solvents (NADES) have been proposed as non-volatile and sustainable solvents, being compatible with both classical and alternative extraction techniques [12] in the recovery of phenols from biomass in general [13,14,15,16,17,18,19], and from grape pomace in particular [14,15,20,21,22,23,24]. Among the several advantages of using natural deep eutectic solvents, such as being able to work at high temperatures without evaporation and being non-toxic for both users and the environment, NADES allow for the tuning of the physical–chemical properties of the solvent, such as viscosity and pH, in order to selectively extract a specific class of compounds [16,17]. Moreover, being composed of a mixture of a hydrogen-bond acceptor (HBA) and a hydrogen-bond donator (HBD), their selectivity can be fine-tuned in the same range of physical–chemical characteristics thanks to the specific intramolecular interactions that occur between the bioactive components to be extracted and the components of the NADES themselves [18].

Since the beginning of research on the valorization of grape pomace, it has been clear to the scientific community that the first parameter-enhancing phenol extraction method involved the pH [19]. Indeed, acidic conditions (especially citric acid) were able, at the same time, to easily destroy the cell membrane, to stabilize anthocyanins, and to promote the hydrolysis of phenolic acid conjugated forms.

A pioneering work by Putnik et al. demonstrated that adding 1% mol of HCl to the extraction solvent had a positive influence on the extraction yields of hydroxycinnamic acids (HCAs), more so than increasing the temperature or prolonging processing times [20,21]. The use of carboxylic acid-based NADES, particularly those with low pH, has been reported to effectively enhance the extraction of phenolic compounds, as these conditions favor the recovery of biomolecules in their neutral form [21]. Recently, acidic NADES formulated with lactic and oxalic acids have shown superior performance compared to their non-acidic counterparts in the extraction of phenolics (anthocyanins, flavonoids, and phenolic acids) particularly when coupled with ultrasound-assisted techniques [22,23].

It is worth noting that the pHs of carboxylic-acid based NADES decrease with increasing temperature [24]. Such an aspect was relatively underestimated in the extraction protocols using NADES as an extraction medium for grape pomace, which have been mainly performed between 45 °C and 65 °C to avoid the degradation of thermolabile compounds such as anthocyanins [22,23].

Usually, a high temperature, typically around 90 °C, is needed to obtain phenolic acids in their free form [25]. It is likely that phenolic acids are stable until 150 °C [26]. On the other hand, it has been demonstrated that carboxylic acid-based DES are stable until temperatures of up to 100 °C, where a small proportion of esterification occurs [27].

In this work, we hypothesized and demonstrated that the combination of high-temperature extraction techniques (90–100 °C) with acidic NADES improves the hydrolysis of phenolic acids from their ester forms and optimizes their selective extraction to obtain them as purified molecules. We made a comparative analysis of five acidic–hydroalcoholic mixtures, with five acidic deep eutectic solvents, under three different extraction techniques, namely solid–liquid, microwave-assisted, and ultrasound-assisted extraction. We evaluated the total phenolic content (TPC), hydroxycinnamic acid content (HCA), and total anthocyanin content (TA) of all samples. A UHPLC-ESI-HRMS quantitative analysis of the phenolic profile, obtained after solvent purification for the best records, was also performed to provide a more reliable solution to exploiting grape pomace as a source of pure phenolic acids. Finally, an ORAC test on the best extraction mixture compared to the purified phytocomplex was performed to clarify the effect of the extraction medium on the antioxidant power against ROS (radical oxygen species). The extract enriched in free phenolic acids could represent a feedstock of active molecules that, after isolation, can serve as building block for the synthesis of pharmaceutical active ingredients against neurodegeneration.

## 2. Materials and Methods

### 2.1. Grape Pomace

Dried grape pomace (residual water content, 7.3%) composed of a blend of Nerello, Merlot, and Cabernet Sauvignon grapes, seeds, and stems was provided by Azienda Agricola De Fazio (Calabria, Italy). Grape pomace was stored under vacuum and used without any selection or pre-treatment. Figure S1 (Supplementary Materials) shows the dimension of the unmodified grape pericarps used for extraction. The calculation of the yields of phenolic compounds was based on grams of dry grape pomace.

### 2.2. Chemicals and Reagents

All chemicals and reagents, namely ethanol, citric acid, oxalic acid, (±) lactic acid, HCl, choline chloride, potassium chloride, sodium acetate, sodium carbonate, Folin–Ciocalteu reagent, AAPH (2,2′-Azobis(2-methylpropionamidine) dihydrochloride), fluorescein sodium salt, phosphate buffer solution, and Trolox were purchased from Merk Italy (Milan, Italy). Purified water was obtained through a Milli-Q Integral 5 system (Millipore, Merck KGaA, Darmstadt, Germany).

Analytical standards of caffeic acid, gallic acid, protocatechuic acid, and trans-ferulic acid were purchased from Merck Italy (Milan, Italy). Working solutions were prepared daily.

### 2.3. Preparation of Hydroalcoholic Solutions

Hydroalcoholic solutions for extraction were prepared by mixing 60% of ethanol with 40% of acidified water. HCl, citric acid, and oxalic acid were used to acidify the water and obtain five solutions (HA) at five different values of final pH: 2.5% *v*/*v* HCl (HA1), 3 g/L citric acid (HA2), 15 g/L citric acid (HA3), 30 g/L citric acid (HA4), 1.4 g/L oxalic acid (HA5). For the pH values of the final solutions, please refer to Table 1 [7].

### 2.4. Preparation of Carboxylic-Acid-Based NADES

The NADES used as extraction solvents were prepared by mixing ChCl and carboxylic acids in the proportions reported in Table 1, under stirring at 80 °C for 2 h. Five different NADES were prepared: ChCl/citric acid 1:1 mol (DES1), ChCl/citric acid 1:2 mol (DES2), ChCl/oxalic acid 1:1 mol (DES3), ChCl/oxalic acid 1:2 mol (DES4), ChCl/lactic acid 1:2 mol (DES5). IR and ^1^H-NMR measurements on the NADES were compared with those reported in the literature (See Supplementary Materials Figures S8–S19) [28,29,30]. A measurement of the pH was performed according to the method reported by Jurić et al. [31]. A total of 20% *w*/*w* of H_2_O was added to freshly prepared NADES before using them as an extraction solvent.

**Table 1:** Chemical composition and pH value of the extraction solvents at 25 °C.

Entry	Solvent	Chemical Composition	Final pH
1	HA1	60%wt EtOH/40% H_2_O with 2.5% HCl	1.5
2	HA2	60%wt EtOH/40% H_2_O with 3 g L^−1^ citric acid	3.4
3	HA3	60%wt EtOH/40% H_2_O with 15 g L^−1^ citric acid	2.8
4	HA4	60%wt EtOH/40% H_2_O with 30 g L^−1^ citric acid	2.5
5	HA5	60%wt EtOH/40% H_2_O with 1.4 g L^−1^ oxalic acid	2.8
6	DES1	ChCl/citric acid 1:1 mol	1.5 ^1^
7	DES2	ChCl/citric acid 1:2 mol	1.0 ^1^
8	DES3	ChCl/oxalic acid 1:1 mol	1.1 ^1^
9	DES4	ChCl/oxalic acid 1:2 mol	1.2 ^1^
10	DES5	ChCl/lactic acid 1:2 mol	1.8 ^1^

^1^ pH value of DES mixtures determined according to the procedure reported in the Ref. [31].

### 2.5. Solid–Liquid Extraction of Grape Pomace with HA Solutions

Hydroalcoholic solutions (HAs) were added to dry grape pomace at a 1:10 solid–liquid ratio, in a Soxhlet apparatus, under stirring at reflux temperature (100 °C) for 2 h. The solid matrix was then removed by filtration under vacuum, washed with water until reaching a final dilution of 1:10 *v*/*v*, and the solution was used without any other purification for spectrophotometric measurements.

### 2.6. Solid–Liquid Extraction of Grape Pomace with NADES Solutions

NADES with 20% *w*/*w* of H_2_O were mixed with dry solid grape pomace with a proportion of 1:10 *w*/*w* and heated at 100 °C under stirring for 2 h. Then, the suspension was cooled to r.t. and centrifuged to separate the solid fraction from the supernatant. The separated solution was then diluted 1:10 *v*/*v* with water, to break the NADES supramolecular interactions, and used for spectrophotometric measurements.

### 2.7. Microwave-Assisted Extraction (MAE) of Grape Pomace

Microwave-assisted extraction (MAE) of grape pomace was realized in a CEM Discover SP oven (Matthews, NC, USA). A total of 1 g of dry pomace was suspended in 10 g of DES3 or DES5 (with the addition of 20% *v*/*v* of H_2_O) in a 30 mL glass tube sealed with a Teflon cup. Microwave heating was performed in dynamic mode at a power of 80 W at temperatures of 65 °C and 100 °C for 10, 30 and 45 min. The extraction solution was then filtered, diluted with water (1:10 *v*/*v*), and used for spectrophotometric measurements.

### 2.8. Ultrasound-Assisted Extraction (UAE) of Grape Pomace

Ultrasound-assisted extraction (UAE) of grape pomace was performed using a titanium high-power US horn by Danacamerini (Turin, Italy). In a glass round-bottomed flask, 1 g of dry pomace was added to 10 g of DES3 or DES5 (with the addition of 20% *v*/*v* of H_2_O) and the flask was immersed in an ice bath to control the temperature in the range of 45–65 °C during extraction. The horn was immersed in the solution and the US was activated (214 KHz, 50 W) for 10, 30, and 45 min. At the end, the extraction mixture was filtered, diluted with water (1:10 *v*/*v*), and used for spectrophotometric measurements.

### 2.9. Total Phenolic Content (TPC)

The total phenolic content (TPC) was measured by Folin–Ciocalteu spectrophotometric assay [32]. Briefly, 1 mL of the extraction solution was combined with 5 mL of Folin–Ciocalteu reagent (diluted 1:10) in a 50 mL graduated flask. After 4 min, 4 mL of Na_2_CO_3_ solution at 7.5% *w*/*v* (15% *w*/*v* for NADES extracts) was added and stirred for 30 min in the dark. The absorbance at 765 nm was recorded in a UV–visible spectrophotometer (UV/VIS Spectrometer Lambda 35, PerkinElmer, Waltham, MA, USA). A calibration curve of gallic acid at concentrations of 50, 100, 150, 250, and 500 mg L^−1^ was used (R^2^ = 0.995, see Figure S2). The total phenolic content was determined as mg of gallic acid equivalents per g (mg GAE g^−1^) of dry pomace.

### 2.10. Hydroxycinnamic Acid Content (HCA)

The total content of hydroxycinnamic acids was determined as previously reported in the literature by Howard et al. [33]. Briefly, 0.25 mL of the extraction solution was mixed with 0.25 mL of a 1 g L^−1^ solution of HCl in aqueous ethanol (96% *v*/*v*) and 4.5 mL of a 2 g L^−1^ solution of HCl (1 M). The absorbance at 320 nm was recorded in a UV–visible spectrophotometer (UV/VIS Spectrometer Lambda 35, PerkinElmer, Waltham, MA, USA). A calibration curve of caffeic acid at concentrations of 1, 2.5, 5, 10, and 25 mg L^−1^ in aqueous methanol (80% *v*/*v*) was used (R^2^ = 0.991, see Figure S3). The HCA content was determined as mg of caffeic acid equivalents per g (mg CAE g^−1^) of dry pomace.

### 2.11. Total Anthocyanin Content (TA)

The total anthocyanin content was determined as previously reported in the literature by Lee et al. [34]. The absorbance of the extraction solutions was determined at two different wavelengths, namely 510 nm and 700 nm, and two different pHs, namely 1.0 and 4.5. Briefly, 1 mL of the extraction solutions was mixed with 9 mL of either a pH 1.0 buffer (0.02 M KCl adjusted with HCl) or a pH 4.5 buffer (0.2 M sodium acetate). The absorbance at both 510 nm and 700 nm was recorded in a UV–visible spectrophotometer (UV/VIS Spectrometer Lambda 35, PerkinElmer, Waltham, MA, USA), and the anthocyanin content was expressed as CGE-cyanidin-3-glucoside equivalent (mg CGE g^−1^ dry pomace) according to the following equation [34]:

$$CGE=\frac{A*M_{w}*DF*V*10^{3}}{\varepsilon *L*M}$$

with

$$A={\left(A_{510}-A_{700}\right)}_{pH1.0}-{\left(A_{510}-A_{700}\right)}_{pH4.5}$$

where

M_w_—molecular weight of cyanidine-3-glucoside (449.2 g mol^−1^);
DF—dilution factor;
V—volume of the extracting solution (L);
M—mass of the solid material (g);
ε—molar extinction coefficient of cyanidine-3-glucoside (26,900 L cm^−1^ mol^−1^);
L—path length (1 cm).

### 2.12. Purification from NADES

DES3 and DES5 extracts obtained by MAE were selected as preferred samples for characterization and phenolic acid quantification by UHPLC-ESI-HRMS, in comparison to the HA2 extract. Before analysis, DES samples were purified from NADES by elution through a Sepabeds Dion HP20 styrere/polyvinylbenzyl resin. Briefly, 10 mL of extract was eluted on a 10 g resin column, activated with 50 mL of ethanol followed by 50 mL of water. ChCl was removed by washing the column with 125 mL of water, then the phenolic acids were recovered washing with 150 mL of ethanol. The ethanolic phase was collected, evaporated under reduced pressure and suspended in 1 mL of EtOH for analysis. In contrast, the HA2 solution was used without any other purification for analysis.

### 2.13. UHPLC-ESI-HRMS Analysis

UHPLC-ESI-HRMS analysis was performed as reported by Roppolo et al. [35]. Briefly, separation was performed by a Dionex Ultimate 3000 RS (Thermo Scientific, Rodano, MI, Italy) equipped with a Hypersil Gold C18 column (100 × 2.1 mm, 1.9 µm particle size). The chromatographic column, maintained at a temperature of 30 °C, was equilibrated in 98% solvent A (0.1% formic acid in ultrapure water) and 2% solvent B (methanol). The concentration of solvent B was linearly increased from 2% to 23% in 6 min, remained in isocratic conditions for 6 min, and finally returned to 2% in 6 min, remaining in isocratic conditions for 3 min. The flow rate was maintained at 300 µL min^−1^. The volume of the injected sample was 5 µL. The total run time, including column wash and equilibration, was 38 min.

A high-resolution Q-Exactive orbitrap mass spectrometer (Thermo Scientific, Rodano, MI, Italy) with an electrospray ionization source, operating in negative mode, was used for detection with the following operating conditions: 70,000 resolving power (defined as FWHM at *m*/*z* 200), IT 100 ms, ACG target = 1 × 10^6^, scan range (100–900 *m*/*z*). MS/MS analysis were performed according to the following operating conditions: resolution, 35.000; AGC target = 1 × 10^5^; maximum IT, 200 ms; collision energy (stepped NCE), 20, 30, 40. The quadrupole isolation window was set to 2.0 *m*/*z*. High-purity nitrogen was used as the sheath gas (30 arb units) and auxiliary gas (10 arb units).

Compounds were characterized according to the corresponding HRMS spectra, retention times, accurate masses, and characteristic fragmentations. Xcalibur software (version 4.1) was used for instrument control, data acquisition, and data analysis.

Individual concentrations of extracted phenolic acids were derived by the external calibration curves of the respective commercial analytical standards. In particular, the concentrations of gallic acid (*m*/*z* 169.0133), syringic acid (*m*/*z* 197.0808), and shikimic acid (*m*/*z* 173.0807) were obtained with respect to a calibration curve of gallic acid (r^2^ = 0.9934, see Figure S4a) in the range between 0.5 and 100 mg L^−1^. The concentrations of protocatechuic acid (*m*/*z* 153.0184), salicylic acid (*m*/*z* 137.023), and benzoic acid (*m*/*z* 121.0284) were obtained with respect to a calibration curve of protocatechuic acid (r^2^ = 0.9984, see Figure S4c) in the range between 0.05 and 100 mg L^−1^. A preset caffeic acid standard calibration curve (r^2^ = 1, See Figure S4b) in the concentration range of 0.01–100 mg L^−1^ was used to determine the content of coumaric acid (*m*/*z* 163.0387). Finally, the dihydroferulic acid (*m*/*z* 195.0287) content was determined using a trans-ferulic acid calibration curve (r^2^ = 0.9955, see Figure S4d) in the range of 0.5–100 mg L^−1^ and 0.5–10 mg L^−1^.

### 2.14. Statistical Analysis

A statistical evaluation for differences was performed on data coming from spectrophotometric assays.

Data were expressed as the mean ± standard deviation (SD) on three repetitions processed by one-way analysis of variance (ANOVA) followed by Tukey’s test for multiple comparisons (GraphPad Prism 10.4.0 scientific software) Tables S1–S3 report the *p*-value for all data statistically evaluated (Supplementary Materials).

### 2.15. ORAC Test

The oxygen radical absorbance capacity (ORAC) test was performed on a microplate fluorometer Varioskan LUX (Thermo Scientific™), controlled by Thermo Scientific™ Skanlt™ Software (Waltham, MA, USA) for microplate readers. The test was performed following the procedure previously published by Nardi et al. [36] for NADES mixtures, on 1 mg mL^−1^ concentrated samples. The final ORAC_FL_ values were expressed as Trolox equivalents (μmol g^−1^ of dry grape pomace) as a mean of three different measurements.

## 3. Results

According to the literature, acid hydrolysis at high temperatures represents a key pre-treatment strategy for the effective release and analysis of phenolic acids and flavonoids present in natural extracts [25]. It has also been demonstrated that increasing the concentration of a strong acid, such as HCl, in the extraction medium, enhances the yield of HCA [24]. Moreover, carboxylic acids are traditionally used both as acid additives in hydroalcoholic solvents [7] and as HBD components in NADES for the extraction of phenolics from grape pomace [22,23]. Considering these assumptions, we decided to undertake a comparative study to evaluate the combined effect of high-temperature extraction (100 °C) with the presence of different carboxylic acids in the extraction medium, either in hydroalcoholic and NADES media. In the following sections, we present our results, collected under traditional and alternative heating, in terms of the total phenolic content (TPC), hydroxycinnamic acid content (HAC), and total anthocyanins content (TA) of all samples. Finally, the UHPLC-ESI-HRMS profiles of the best extraction procedures after purification are presented and the phenolic acids are quantified.

### 3.1. Solid–Liquid Extraction

Our study started with the comparison of five acidic hydroalcoholic solutions (HA1-5) and five carboxylic-acid-based deep eutectic solvents (DES1-5), under traditional heating, as acidic extraction medium. Table 1 shows the chemical composition and the pH of the solvents used for the Soxhlet solid–liquid extraction at 100 °C of dry grape pomace.

HA solutions were formulated using HCl, citric acid (at different concentrations), and oxalic acid, resulting in pH values ranging from 1.5 to 3.4. On the other hand, acidic DES were prepared by mixing ChCl with different molar ratios of citric, oxalic, and lactic acid, yielding pH values between 1.0 and 1.8. These mildly-to-strongly acidic media were compared on their ability to enhance the extraction of the phenolic acid fraction. Extraction efficiency was evaluated by determining the total phenolic content (TPC), hydroxycinnamic acid content (HAC), and total anthocyanins content (TA) by spectrophotometric assays. A solid–liquid ratio of 1:10 was adopted for all extraction tests. Three independent replicates of each experiment were carried out. The results are collected in Table 2.

As expected, citric acid proved to be the most effective acid for HA solutions (entries 2–4, Table 2). However, our results clearly showed that the phenolic content in general, and the HCA in particular, are more influenced by the nature of the acid than the pH of the solution. This trend is evident when comparing results obtained with HA3 and HA5 (entries 3 and 5, Table 2) and even more so when comparing all acidic NADES with HA1. Despite having similar pH values, solvents obtained using a carboxylic acid instead of HCl gave the best results. Among the NADES, DES3 and DES5, composed of ChCl/oxalic (1:1) and ChCl/lactic (1:2) acids, showed the best extraction performance in terms of both TPC and HCA (entries 8 and 10, Table 2). Interestingly, increasing concentrations of citric acid in HA enhanced anthocyanin extraction (entry 4, Table 2), whereas acidic NADES appeared to negatively affect the recovery of these bioactive components.

### 3.2. Alternative Heating Extraction

DES3 and DES5, which were the best solvents in the traditional extraction of phenolic acids from grape pomace, were selected to explore the dependence on the heating method. Microwave-assisted extraction (MAE) and ultrasound-assisted extraction (UAE) of dry grape pomace were performed by suspending grape pomace in DES3 and DES5 at the same solid–liquid ratio used in traditional extraction. Three independent replicates of each experiment were carried out. The spectrophotometric results obtained by MAE and UAE at different temperatures and times are presented in Table 3. Notably, to avoid sudden increases in temperature due to the high-temperature hot spot phenomena in the medium, we decide to control the temperature by putting the sample in an ice/water bath, thus obtaining a working temperature ranging between 60 and 65 °C. For a better comparison, the temperature settings for MAE were both 65 °C and 100 °C.

The results were statistically compared to the corresponding solid–liquid extraction procedure, reported in Table 2 and used as a control. While the TPC was strongly influenced by the heating source, in any case, the HCA content was significantly different from that in the classical solid–liquid extraction. Our results demonstrated that MAE at 100 °C for 10 min gave rise to the highest yields of HCA. Indeed, MAE was comparable to classical solid–liquid extraction in terms of extraction yields at the same temperature for DES3, while a drastic improvement was registered for DES5. In both cases, the extraction took much shorter times (entries 4 and 8, Table 3), thus providing an advantage in terms of energy consumption. Nevertheless, such extraction methods generated the degradation of anthocyanins and prolonged times resulted in carbonization of the natural matrix. In contrast, UAE at medium temperatures did not provide any real improvements.

### 3.3. UHPLC-ESI-HRMS Characterization of Extracts

**Figure 2:**
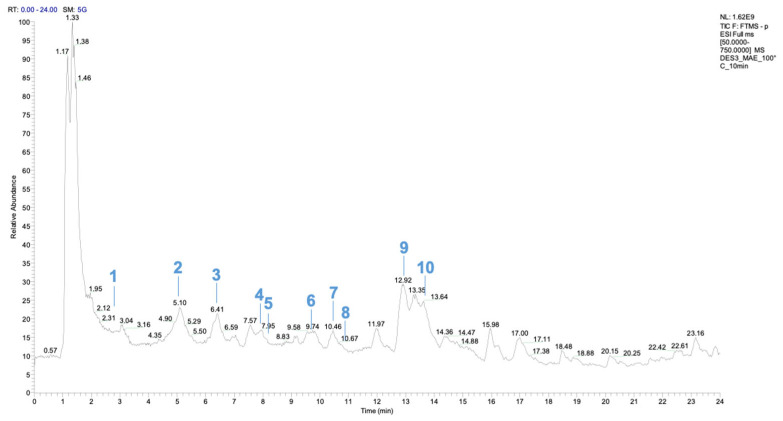
Full scan chromatogram obtained by UHPLC-ESI-HRMS for the DES3_MAE_100 °C_10 min sample, highlighting the identified phenolic acids only, as reported in Table 4.

According to the comparative study on the best extraction conditions, MAE with DES3 and DES5 gave rise to the highest yield of HCA in the shortest time. Consequently, these two extracts were both selected for the LC-MS characterization and quantification of phenolic acids, in comparison to hydroalcoholic extraction (HA2). Concerning DES, sample preparation was performed by elution through a vinyl/polystyrene resin [22], able to release ChCl by washing in water and to retain phenolic acids until the next EtOH elution was performed. The recovery was determined by spectrophotometric assays on the eluted phase (60% and 75% for DES3 and DES5, respectively). The full scan chromatogram obtained for the DES3 sample is shown in Figure 2, highlighting the identified phenolic acids reported in Table 4.

The list of compounds identified in the considered samples, grouped according to their chemical classification, are reported in the following table.

A total of 29 compounds, including several phenolic acids, were identified using ESI negative mode (Figure 2). In general, the molecule variability in NADES samples is higher than in hydroalcoholic solution. In the MS^2^ spectra of gallic acid (compound **1**), protocatechuic acid (compound **2**), and salicylic acid (compound **3**) [37], the predominant daughter ions were originated by the loss of 44 Da, corresponding to the carbon dioxide group [M-H-CO_2_]^−^, a typical fragment of phenolic acids. The compound **4** at *m*/*z* 121.0284 was identified as benzoic acid, showing a characteristic fragmentation pattern of simple benzoic acid. The compound **5** with [M-H]^−^ ion at *m*/*z* 195.0287 was identified as dihydroferulic acid, considering the presence of its characteristic fragment ion at *m*/*z* 151.0387 due to the loss of carbon dioxide and *m*/*z* 123.0436 after the additional loss of the methyl group [38]. The presence of a p-hydroxybenzoic acid derivative (compound **6**) with *m*/*z* 165.0184, was confirmed by the daughter ion at *m*/*z* 121.028, as already affirmed by Rini Vijayan et al. [39]. Ethyl gallate (compound **7**) at *m*/*z* 197.0444 with daughter ions at *m*/*z* 169.0129 and *m*/*z* 125.0229 suggested the loss of an ethyl group from an esterified one [40]. The compound **8** at *m*/*z* 165.0544 and daughter ions at *m*/*z* 147.0437 and 119.0487 were identified as dihydro-3-coumaric acid [41]. The mass signal at *m*/*z* 197.0808 was recognized as syringic acid (compound **9**) [42]. Coumaric acid (compound **10**) at *m*/*z* 163.0387 was identified based on its fragmentation pattern characterized by the loss of a carbon dioxide group (44 Da), as reported by Myrtsi et al. [43]. Finally, among the biosynthetic precursors of phenolic acid, the presence of shikimic acid (compound **25**) at *m*/*z* 173.0807 was confirmed by the daughter ions at *m*/*z* 129.0907 and *m*/*z* 155.0707.

Among flavonoids, dihydromyricetin (compound **11**) at *m*/*z* 319.0452 was found comparing its fragmentation pattern with the literature [44]. The compound **12** at *m*/*z* 303.0503 was identified as taxifolin (or dihydroquercetin). The MS^2^ spectra showed the characteristic fragment at *m*/*z* 285.0396, 193.0130, and 177.0180 as observed by Escobar-Avello et al., 2019 [45]. The compound **13** was identified as kaempferol as observed by Queralt et al. [46]. The compound **14** at *m*/*z* 477.0679 was identified as quercetin-7-O-glucuronic acid. The MS^2^ spectrum showed a predominant ion [M-H-176]^−^ at *m*/*z* 301.0357 due to the loss of a glucuronic unit, and the characteristic fragments at *m*/*z* 255.178 and 151 of quercetin aglycone, as observed by Li et al. [47]. Concerning the MS^2^ spectrum of compound **15**, with the daughter ions at *m*/*z* 179.0342 and 15.0026, it displayed the typical fragmentation of quercetin [46]. Among flavan-3-ols, catechin (compound **16**) at *m*/*z* 289.0719 and fragment ions at *m*/*z* 245.0818 and 179.0341 were identified by comparison with the commercial standard. The compound **17** with [M-H]^−^ ion at *m*/*z* 441.0823 was identified as epicatechin gallate, considering the presence of its characteristic fragment ion at *m*/*z* 289.0719 [45]. Moreover, one anthocyanin was identified as deprotonated ions. The compound **18** was recognized as chalcone delphinidin, with MS^2^ fragments at *m*/*z* 183.0287 and *m*/*z* 153.0180 [48]. Furthermore, other organic compounds (compound **19**–**29**) were identified.

Among them, the HBD component of the corresponding NADES was found; indeed, it shared the same functional group with the desired analyte, and it was released by the resin after washing with ethanol. In this regard, while oxalic acid (compound **20**) at *m*/*z* 89.0229 represented a minor compound in the DES3 extract (see Figure 2), many lactate derivatives were identified in the DES5 extract (compounds **21**, **23**, and **26**–**29**) due to the spontaneous polymerization of lactic acid (see Figure S5). Unfortunately, the amount of such compounds was significantly higher than the other phenolic compounds found in the DES5 extract. We decided to perform a blank test on DES5 in order to establish whether such compounds could interfere with the HCA determination. In more detail, DES5, without any natural matrix, was processed under the same heating conditions of the MAE extraction procedure and the resulting mixture was subjected to UHPLC-UV-ESI-HRMS characterization. As expected, the HRMS profile of the blank sample exactly fitted the DES5 extraction sample (see Figure S6), except for the sub-spectrum due to the phenolic compounds (see Figure S5b). On the other hand, the comparison of UV spectra, registered at 330 nm, showed a significant absorbance baseline in both the blank sample and in the extract (see Figure S7). This result suggested that the presence of lactate derivatives could interfere with the spectrophotometric measurement of total HCA in DES5 extracts.

### 3.4. UHPLC-ESI-HRMS Quantification of Phenolic Acids

Phenolic acids identified by HRMS in the DES3, DES5, and HA2 extracts were quantified by external calibration curves of selected standards as reported in Section 2.12.

The results, given as mg of phenolic acid per g of grape pomace, are summarized in Table 5.

Notably, the total phenolic acid contents determined by HRMS quantification were consistently lower than those estimated by spectrophotometric measurements, for both samples. In addition to the partial recovery of the sample, this result is clearly due to the interference of other classes of compounds absorbing at similar wavelengths (i.e., flavonoids and flavanols) in the spectrophotometric assays. Moreover, Table 5 shows that, conversely to the total HCA spectrophotometric determination, the DES3 sample is richer in phenolic acids with respect to the DES5 sample, thus demonstrating that the unexpected and undesired polymerization of lactic acid is responsible for the increased absorbance at 320 nm in DES5 samples with respect to DES3. Both DES3 and DES5 samples were richer in phenolic acids compared to HA2.

### 3.5. ORAC Test

One of the advantages of NADES extraction techniques lies in the intrinsic non-toxicity of their components, which allows the resulting extracts to be directly used as antioxidant phytocomplex without further purification [49,50]. To prove the effect of DES3 medium on phenolics antioxidant power against ROS, we performed the ORAC (oxygen radical absorbance capacity) test on three different samples: DES3_blank_, composed of DES3 alone; DES3_mixture_, composed of the diluted extraction mixture obtained after MW heating; DES3_extract_, composed of the isolated phytocomplex obtained after resin purification. The results are reported in Table 6:

The samples were analyzed at the same concentration (1 mg mL^−1^). The antioxidant power of DES3_blank_ was negligible, as previously reported in the literature [49]. DES3_mixture_, obtained by the MW-assisted extraction of grape pomace, showed a good antioxidant power, with ORAC values higher than hydroalcoholic extracts of the same natural matrix having the same TPC [51]. This result is not surprising, as it was already reported that NADES could stabilize phenols and improve their antioxidant power, even if they do not have any antioxidant activity themself [36,50]. It would be expected that the purified extract (DES3_extract_), free from the 90% of inactive DES3, showed an important increase in the antioxidant power. Nevertheless, our results reported only a slight increase in this value, thus demonstrating that NADES components, even if not yet coordinated in NADES after dilution, could have an influence on the antioxidant activity of the bioactive molecules of the sample.

## 4. Discussion

Phenolic acids are present in grape pomace in a conjugate form; their extraction needs acidic hydrolysis to be efficient [24,25]. In our work, we explored different acidic extraction media, both hydroalcoholic solutions and NADES, in combination with alternative energy sources, for the in situ hydrolysis and extraction of grape pomace, with the aim of optimizing the yield of phenolic acids. In the preliminary part of the work, a comparative study was performed to understand the dependence of phenolic acid extraction efficiency on pH and on the nature of the acid. Our results clearly indicate that, although strongly acidic conditions are essential to hydrolyze and extract phenolic acids, the chemical nature of the acid showed an even greater influence than pH alone in determining the selectivity and efficiency of extraction. Specifically, it was found that carboxylic acids are more efficient than HCl at the same pH values, with citric acid proven most effective for the extraction of anthocyanins, while lactic and oxalic acid showed a superior performance for phenolic acid recovery. Indeed, we found that oxalic- and lactic-acid-based DES, in combination with microwave irradiation, were able to efficiently hydrolyze and extract phenolic acids. For instance, the extraction of grape pomace with ChCl/oxalic acid 1:1 DES, heated for 10 min at 100 °C, under microwave irradiation, produced a phytocomplex containing, in addition to other phenols, 1.15 mg g^−1^ of phenolic acids. This result is significantly higher than those reported in the literature. Indeed, classic solid–liquid extractions with hydroalcoholic solutions, acidified with both organic or inorganic acids, have produced an amount of phenolic acids two orders of magnitude lower than our method [20]; on the other hand, both US- and MW-assisted methods using non-acidic DES have extracted phenolic acids in the range of 2.74–160 μg g^−1^ [14,15]. Among the few comparable examples, the work of Alrugah et al. [25] employing a non-acidic ternary DES under US assistance (1 h, 65 °C) also reported high percentages of phenolic acids from grape pomace. However, their quantification relied on HPLC-UV following sample pre-treatment consisting of acidic hydrolysis, which increased the amount of free phenolic acids. No data were reported on the amount of free phenolic acids after extraction.

Regarding acidic NADES, lactic-acid-based DES under US assistance have been reported in the literature as the best solvents for phenolic acid recovery [23], ranging between 0.7 and 1.0 mg g^−1^. Nevertheless, our study was the first report employing UHPLC-ESI-HRMS as a quantification method to support spectrophotometric assays. We reported here that lactic acid NADES underwent the spontaneous polymerization of lactic acid and such polymers cannot be separated by the natural extract using the styrene/polyvinyl resin purification, as suggested for the separation of natural extracts from NADES media [22]. Indeed, UHPLC-ESI-HRMS analysis revealed that the final phytocomplex is dirty, due to the presence of such polymers, which had a non-zero absorbing power at 320–330 nm, thus interfering with spectrophotometric determination. Moreover, HPLC-UV methods working at different wavelengths (254, 370, 560 nm), often reported in the literature [22,23] for phenolic acid quantification, were not able to detect such polymers, thus inducing an error in the interpretation of the experimental data referred of lactic-acid-based NADES. Some studies reported the temperature instability of lactic acid based NADES, even for low temperatures [27]. Despite these findings, our work demonstrated that, independently from the heating source and the temperature of extraction, lactic acid based NADES are not the most suitable media for natural matrix extraction, due to their thermal instability; moreover, spectrophotometric assays and HPLC-UV quantifications are not the most reliable and exhaustive methods to characterize such extracts.

Concerning the alternative heating sources explored in this work, microwave irradiation (MAE), at a high temperature (100 °C) for a short time (10 min) was the most efficient within our scope. Indeed, MAE allowed us to reach comparable yields with respect to classical solid–liquid extraction in shorter time, thus resulting energetically more sustainable. Nevertheless, prolonged time of exposure was detrimental for the phytocomplex. In our study, UAE performed slightly less well than MAE as a technique to obtain free phenolic acids after acid hydrolysis. Indeed, UAE was often reported in the literature as the softest technique to extract temperature-labile molecules without breaking their structure [22,23].

Finally, it is necessary to briefly discuss the ecotoxicity and human toxicity of such NADES. According to the literature, the eco-compatibility of NADES against organic solvents is due to their low volatility, but the final disposal of NADES and the toxicity of their single components is controversial. It has been established that ChCl itself is non-toxic [52], while ChCl/carboxylic acid NADES have moderate toxicity against Gram-negative and Gram-positive bacteria [53,54], marine bacteria [55], and eukaryotic cells [49]. Such low toxicity is mainly correlated with the carboxylic acid, with EC_50_ values comparable to those of the single component and dependent on the acid content. In more detail, considering the carboxylic acids used in this study, the order of toxicity was lactic acid < citric acid < oxalic acid. The acid character is responsible for protein denaturation, and consequent activation of enzymatic pathways leading to cell death [49,52,53,54,55]. On the other hand, low-to-negligible phytotoxicity was reported for all carboxylic-acid-based NADES [49].

Concerning the specific phenolic acids present in the grape pomace extract, dihydro-3-coumaric acid (420 μg g^−1^) was the most representative, followed by gallic acid (308 μg g^−1^). More generally, we found that hydroxybenzoic acids were more representative than hydrocinnamic acids. Further studies to obtain such phenolic acids as isolated molecules are needed.

On the other hand, the possibility of using the DES3 extract as a “ready-to-use” antioxidant formulation was demonstrated by the ORAC test, performed on the phenolic extract with and without NADES. This is a standardized test usually applied to measure the ROS chain-breaking activity of food and beverages, by H atom transfer. Catecholic compounds, such as phenolic acids, are particularly reactive, as they can originate resonant stable radicals after hydrogen transfer, due to the intramolecular hydrogen interaction between the *o*-diphenolic compounds [56].

Indeed, our results confirmed that, even if the DES3 itself has no activity against ROS, the formulation of phenols in the DES had an antioxidant activity higher than hydroalcoholic extracts with comparable TPC values, previously reported in the literature [50]. Although, after dilution, our extraction mixture cannot still be considered a NADES, this result revealed a role of NADES components in the antioxidant power of the mixture. Potential applications of ChCl/oxalic acid 1:1 NADES as a food preservative or antioxidant active ingredient in pharmaceutical formulations have been investigated in the literature [49]. As mentioned in a previous study, it showed medium toxicity against HeLa and MCF-7 cells (EC_50_ of 330.90 ± 29.75 and 558.98 ± 54.32 mg L^−1^, respectively), while negligible toxicity was found in normal HEK293Tcells (EC_50_ > 2.000 mg L^−1^). Moreover, antimicrobial and antibacterial activity against several Gram-positive and Gram-negative bacteria and yeast was found, due to the presence of oxalic acid [49]. Based on these preliminary results, the potential applications of the DES3 extraction mixture as a “ready-to-use” preparation in food preservation, especially for those applications exploiting its antibacterial power, need to be further explored. Other antioxidant activity assays, both chemical (DPPH, ABTS, and FRAP assays) and cellular (H_2_O_2_-induced ROS inhibition in cell culture), need to be performed.

In addition, we demonstrated that the method provides, after purification from NADES, an extract enriched in phenolic acids with improved antioxidant activity relative to the simple formulation. Future work will focus on optimizing separation techniques to obtain the single components of the extract. Single phenolic acids will be used as chemical building blocks for the synthesis of nature-inspired active ingredients against neurodegeneration.

## 5. Conclusions

The present work demonstrated that ChCl/oxalic acid 1:1 NADES, combined with MAE at 100 °C, is the best medium for the in situ hydrolysis and extraction of phenolic acids from grape pomace. The extract was fully characterized and the yield of total phenolic acids, determined by UHPLC-ESI-HRMS, was one order of magnitude higher than previous methods reported in the literature. Notably, the nature of the acidic component of the DES is crucial in selecting the family of molecules to be extracted and it can be tailored to diverse extents. Moreover, the NADES reaction medium can stabilize and potentiate the antioxidant power of the mixture.

## Figures and Tables

**Table 2:** TPC, HAC, and TAC values of grape pomace extract obtained with different extracting solvents by traditional heating ^1^.

Entry	Solvent	TPC ^3^ (mg GAE g^−1^)	HCA ^3^ (mg CAE g^−1^)	TA ^3^ (mg CGE g^−1^)
1	HA1	23.57 ± 0.48 ^a^	2.76 ± 0.7 ^a^	0.18 ± 0.01 ^a^
2	HA2	21.74 ± 0.69 ^abc^	3.21 ± 0.04 ^ab^	0.18 ± 0.02 ^a^
3	HA3	20.31 ± 1.90 ^bc^	2.87 ± 0.16 ^ab^	7.75 ± 0.05 ^b^
4	HA4	19.06 ± 0.68 ^bc^	2.68 ± 0.03 ^a^	15.99 ± 0.10 ^c^
5	HA5	18.40 ± 0.41 ^c^	2.41 ± 0.24 ^a^	8.32 ± 0.01 ^b^
6	DES1 ^2^	39.56 ± 2.20 ^d^	4.73 ± 0.06 ^a^	0.65 ± 0.16 ^a^
7	DES2 ^2^	32.01 ± 1.97 ^e^	3.35 ± 0.34 ^a^	1.21 ± 0.12 ^a^
8	DES3 ^2^	62.61 ± 0.84 ^f^	6.05 ± 0.26 ^b^	2.68 ± 0.10 ^a^
9	DES4 ^2^	36.40 ± 0.64 ^d^	1.36 ± 0.08 ^ac^	1.09 ± 0.03 ^a^
10	DES5 ^2^	53.35 ± 1.41 ^g^	5.37 ± 0.23 ^ad^	0.28 ± 0.11 ^a^

^1^ Extraction conditions: 1:10 solid–liquid ratio, Soxhlet extraction, 100 °C, 2 h. ^2^ 20% *v*/*v* of water was added before extraction. ^3^ Data are reported as the mean ± SD of three independent experiments Different lowercase letters indicate statistically significant comparisons (*p* < 0.001).

**Table 3:** TPC, HAC, and TAC values of grape pomace extract obtained with DES3 and DES5 solvents by alternative heating ^1^.

Entry	Solvent ^2^	Heating Method	T (°C)	Time (min)	TPC ^4^ (mg GAE g^−1^)	HCA ^4^ (mg CAE g^−1^)	TA ^4^ (mg CGE g^−1^)
1	DES3	MAE	65	10	36.27 ± 4.21 ^a^	5.58 ± 0.11	3.73 ± 0.05
2	DES3	MAE	65	30	24.60 ± 4.20 ^b^	4.04 ± 0.12	2.32 ± 0.02
3	DES3	MAE	65	45	32.53 ± 1.86 ^c^	4.91 ± 0.25	2.45 ± 0.12
4	DES3	MAE	100	10 ^3^	45.64 ± 1.15 ^d^	5.84 ± 0.03	0.28 ± 0.03
5	DES5	MAE	65	10	46.97 ± 10.19 ^e^	4.29 ± 0.13	0.04 ± 0.12
6	DES5	MAE	65	30	26.66 ± 6.56 ^f^	3.30 ± 0.04	0.03 ± 0.03
7	DES5	MAE	65	45	43.75 ± 3.36 ^e^	4.80 ± 0.13	0.09 ± 0.13
8	DES5	MAE	100	10 ^3^	32.20 ± 3.64 ^e^	9.81 ± 0.19	-
9	DES3	UAE	60–65	10	37.12 ± 0.76 ^bc^	4.18 ± 0.15	0.51 ± 0.09
10	DES3	UAE	60–65	30	35.92 ± 0.04 ^bc^	2.79 ± 0.12	-
11	DES3	UAE	60–65	45	37.86 ± 0.50 ^bc^	3.86 ± 0.08	-
12	DES5	UAE	60–65	10	38.78 ± 1.64 ^e^	3.66 ± 0.02	-
13	DES5	UAE	60–65	30	35.46 ± 0.86 ^e^	3.29 ± 0.06	-
14	DES5	UAE	60–65	45	42.29 ± 3.36 ^e^	5.24 ± 0.21	-

^1^ 1:10 solid–liquid ratio. ^2^ 20% *v*/*v* of water was added before extraction. ^3^ Longer times gave rise to decomposition of the extraction mixture. ^4^ Data are reported as the mean ± SD of three independent experiments. Different lowercase letters indicate statistically significant comparisons (*p* < 0.001) with each other and with respect to the control (entries 8 and 10, Table 2).

**Table 4:** UHPLC_ESI_HRMS identification of phenolic compounds of grape pomace obtained by microwave-assisted extraction with DES3 and DES5, in comparison to HA2 extract.

Compounds N°	Compound	R.T.	[M-H]^−^ (*m*/*z*)	[2M-H]^−^ (*m*/*z*)	Fragment Ion (*m*/*z*)	Extract
Phenolic acids						
**1**	Gallic acid	2.69	169.0133	-	125.0232	DES3, DES5, HA2
**2**	Protocatechuic acid	4.88	153.0184	-	109.0283	DES3, DES5, HA2
**3**	Salicylic acid	6.41	137.023	-	93.033	DES3, DES5, HA2
**4**	Benzoic acid	7.95	121.0284	-	-	DES3, DES5, HA2
**5**	Dihydroferulic acid	7.95	195.0287	-	151.0387; 123.0436	DES3, DES5
**6**	p-Hydroxybenzoic acid derivative	9.73	165.018	-	121.028	DES3, DES5
**7**	Ethyl gallate	10.49	197.0444	-	169.0129; 125.0229	DES3, DES5, HA2
**8**	Dihydro-3-coumaric acid	10.58	165.0544	-	147.0437; 119.0487; 121.0280	DES3
**9**	Syringic acid	12.92	197.0808	395.1701	153.0907	DES3, DES5
**10**	Coumaric acid	13.72	163.0387	-	119.0487	DES3, DES5
Flavonols						
**11**	Dihydromyricetin	8.23	319.0452	-	301.0346; 193.0131; 125.0229	DES3, DES5
**12**	Taxifolin	9.85	303.0503	-	285.0396; 193.0130; 177.0180; 167.0336; 125.0229; 109.0279	DES3
**13**	Kaempferol	16.98	285.0399	-	257.0449; 125.0229; 163.0023; 217.0497; 257.0449	DES3
**14**	Quercetin-7-O-glucuronic acid	17.17	477.0679	-	301.0357; 178.9979; 151.0027; 135.0079	DES5, HA2
**15**	Quercetin	20.15	301.0354	603.077	178.9978; 151.0026; 121.0283; 107.0125; 273.0405	DES3, DES5
Flavan-3-ols						
**16**	Catechin	7.75	289.0719	579.1512	245.0818; 203.0708, 179.0341; 151.0390; 125.0233; 109.0282; 104.9904; 97.0282	DES5
**17**	Epicatechin gallate	13.42	441.0823	881.1573	289.0718; 245.0819; 169.0134; 151.0390; 125.0232	DES5
Anthocyanins						
**18**	Chalcone delphinidin	13.54	319.045	-	183.0287; 153.0180; 139.0387;	DES3, DES5
Others						
**19**	Tartaric acid	1.17	149.0081	-	103.0024; 87.0074; 72.9917; 59.0125	DES3, DES5
**20**	Oxalic acid	1.18	89.0229	-	60.9916	DES3
**21**	Lactate derivative 1	5.30	161.0441	-	89.0228	DES5
**22**	Isopropylmalic acid	7.6	175.0604	-	157.0497; 131.0703; 115.0388; 113.0596; 85.0645	DES3, DES5
**23**	Lactate derivative 2	9.35	233.0657	-	161.0441; 89.0228	DES5
**24**	Unknown	13.31	171.0651	-	127,075	DES3, DES5
**25**	Shikimic acid	16.08	173.0807	-	129.0907; 155.0707	DES3, DES5, HA2
**26**	Lactate derivative 3	16.20	305.0871	-	161.0441; 89.0228	DES5
**27**	Lactate derivative 4	19.01	377.1081	-	161.0441; 89.0228	DES5
**28**	Lactate derivative 5	20.33	449.1291	-	161.0441; 89.0228	DES5
**29**	Lactate derivative 6	21.11	521.1505	-	161.0441; 89.0228	DES5

**Table 5:** UHPLC-ESI-HRMS quantification of phenolic acids.

Entry	Phenolic Acid	HA2 (mg g^−1^) ^1^	DES3 (mg g^−1^) ^1^	DES5 (mg g^−1^) ^1^
1	Gallic acid	0.0472 ± 0.0023	0.3086 ± 0.0093	0.0505 ± 0.0015
2	Protocatechuic acid	0.0618 ± 0.0017	0.0770 ± 0.0021	0.1460 ± 0.0045
3	Salicylic acid	0.0039 ± 0.0007	0.0853 ± 0.0016	0.0636 ± 0.0017
4	Benzoic acid	0.0061 ± 0.0005	0.0050 ± 0.0003	trace
5	Dihydroferulic acid	trace	0.0119 ± 0.0008	0.0046 ± 0.0007
6	p-Hydroxybenzoic acid derivative	trace	0.0397 ± 0.0014	0.1062 ± 0.0037
7	Ethyl gallate	0.0126 ± 0.0013	0.0348 ± 0.0009	0.0116 ± 0.0006
8	Dihydro-3-coumaric acid	trace	0.4249 ± 0.0072	0.3008 ± 0.0059
9	Syringic acid	trace	0.0585 ± 0.0013	0.0778 ± 0.0021
10	Coumaric acid	trace	0.0107 ± 0.0004	0.0067 ± 0.0005
Total		0.1315 ± 0.0065	1.1565 ± 0.0253	0.7678 ± 0.0275

^1^ Data are reported as the mean ± SD of three independent experiments.

**Table 6:** Oxygen radical absorbance capacity (ORAC) values of DES3 extract.

Sample	Concentration (mg mL^−1^)	ORAC_FL_^a^
DES3_blank_	1	-
DES3_mixture_	1	3857 ± 89
DES3_extract_	1	5218 ± 162

^a^ ORAC values expressed as µmol Trolox/gr of dry grape pomace. The reported values are the mean ± SD of three independent measurements.

## Data Availability

The data presented in this study are available on request from the corresponding author.

## Supplementary Materials

The following supporting information can be downloaded at https://www.mdpi.com/article/10.3390/antiox14060643/s1, Figure S1. Dimension of grape pericarps; Figure S2. Correlation of the peak area obtained by UV–visible spectrophotometer and analytical standard concentration of gallic acid for the determination of total phenolic content; Figure S3. Correlation of the peak area obtained by UV–visible spectrophotometer and analytical standard concentration of caffeic acid for the determination of hydroxycinnamic acid content; Figure S4. Correlation of the peak area obtained by UHPLC-ESI-HRMS and analytical standard concentration of gallic acid (a); caffeic acid (b); protocatechuic acid (c); trans-ferulic acid (d); Figure S5. Full scan chromatogram obtained by UHPLC-ESI-HRMS for the DES5_MAE_100 °C_10 min sample, highlighting the products of a spontaneous polymerization of lactic acid (a) and zoom of sub-spectrum (b); Figure S6. Comparison between full scan chromatograms obtained by UHPLC-ESI-HRMS for the DES5_BLK sample (a) and DES5_MAE_100 °C_10 min sample (b); Figure S7. Comparison between chromatograms obtained by UHPLC-UV/VIS (330 nm) for the DES5_BLK sample (a) and DES5_MAE_100 °C_10 min sample (b); Figure S8. FL fluorescence decay curve induced by AAPH; Figure S9. Linear plot of AUC vs. Trolox concentrations; Figure S10. FT-IR spectrum of DES1; Figure S11. ^1^H-NMR spectrum of DES1; Figure S12. FT-IR spectrum of DES2; Figure S13. ^1^H-NMR spectrum of DES2; Figure S14. FT-IR spectrum of DES3; Figure S15. ^1^H-NMR spectrum of DES3; Figure S16. FT-IR spectrum of DES4; Figure S17. ^1^H-NMR spectrum of DES4; Figure S18. FT-IR spectrum of DES5; Figure S19. ^1^H-NMR spectrum of DES5; Table S1. Tukey’s multiple comparisons test on Table 2; Table S2. Tukey’s multiple comparisons test on Table 3 (against DES3); Table S3. Tukey’s multiple comparisons test on Table 3 (against DES5).

## Abbreviations

The following abbreviations are used in this manuscript:

MAE	Microwave-assisted extraction
UAE	Ultrasound-assisted extraction
MW	Microwave
US	Ultrasound
NADES	Natural deep eutectic solvent
DES	Deep eutectic solvent
HBA	Hydrogen-bond acceptor
HBD	Hydrogen-bond donator
HCA	Hydroxycinnamic acid
TPC	Total phenolic content
TA	Total anthocyanins
GAE	Gallic acid equivalents
CAE	Caffeic acid equivalents
CGE	Cyanidin-3-glucoside equivalents
ORAC	Oxygen radical absorbance capacity
