# Supplementary material for: Combining Carboxylic-Acid-Based Deep Eutectic Solvents and High Temperatures Enhances Phenolic Acid Extraction from Grape Pomace
Source: Antioxidants (Basel). 2025 May 27;14(6):643. doi: 10.3390/antiox14060643 (PMC12189704; doi:10.3390/antiox14060643)
Supplement: Supplementary file 1 [file antioxidants-14-00643-s001.zip › antioxidants-3603839-supplementary.pdf]

SUPPLEMENTARY MATERIALS

Combining Carboxylic-Acid-based Deep Eutectic Solvents and High Temperatures Enhances Phenolic Acid Extraction from Grape Pomace

Francesca Lorenzo †, Marialaura Frisina †, Sonia Bonacci, Monica Nardi, Manuela Oliverio * and Antonio Procopio

Figure S1. Dimension of grape pericarps. pag.3

Figure S2. Correlation of the peak area obtained by UV-visible spectrophotometer and analytical standard concentration of gallic acid for the determination of total phenolic content. pag. 4

Figure S3. Correlation of the peak area obtained by UV-visible spectrophotometer and analytical standard concentration of caffeic acid for the determination of hydroxycinnamic acids content. pag. 4

Figure S4. Correlation of the peak area obtained by UHPLC-ESI-HRMS and analytical standard concentration of gallic acid (a); caffeic acid (b); protocatechuic acid (c); trans-ferulic acid (d). pag. 5

Figure S5. Full Scan chromatogram obtained by UHPLC-ESI-HRMS for the DES5_MAE_100°C_10 min sample, highlighting the products of a spontaneous polymerization of lactic acid (a) and zoom of sub-spectrum (b*).* *pag. 6*

Figure S6. Comparison between Full Scan chromatograms obtained by UHPLC-ESI-HRMS for the DES5_BLK sample (a) and DES5_MAE_100°C_10 min sample (b). pag. 7

Figure S7. Comparison between chromatograms obtained by UHPLC-UV/VIS (330nm) for the DES5_BLK sample (a) and DES5_MAE_100°C_10 min sample (b). pag. 7

Figure S8. FL fluorescence decay curve induced by AAPH. pag. 8

Figure S9. Linear plot of AUC vs Trolox concentrations. pag. 8

Table S1. Tukey's multiple comparisons test on Table 2 pag. 9

Table S2. Tukey's multiple comparisons test on Table 3 (against DES3) pag. 11

Table S3. Tukey's multiple comparisons test on Table 3 (against DES5) pag. 12

Figure S10. FT-IR spectrum of DES1. pag. 15

Figure S11. 1H-NMR spectrum of DES1. pag. 16

Figure S12. FT-IR spectrum of DES2. pag. 17

Figure S13. 1H-NMR spectrum of DES2. pag. 18

Figure S14. FT-IR spectrum of DES3. pag. 19

Figure S15. 1H-NMR spectrum of DES3. pag. 20

Figure S16. FT-IR spectrum of DES4. pag. 21

Figure S17. 1H-NMR spectrum of DES4. pag. 22

Figure S18. FT-IR spectrum of DES5. pag. 23

Figure S19. 1H-NMR spectrum of DES5. pag. 24

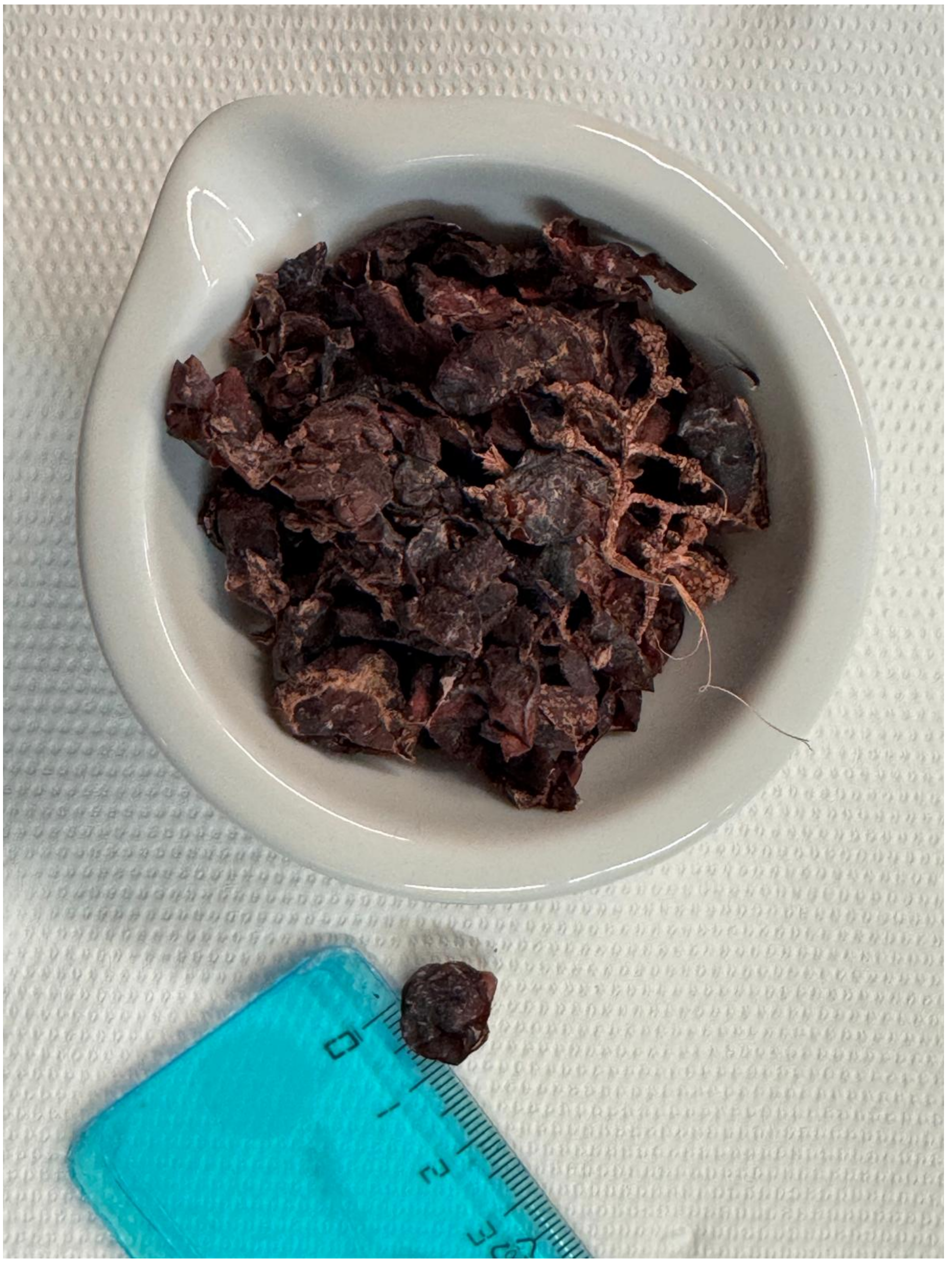

Figure S1. *Dimension of grape pericarps.*

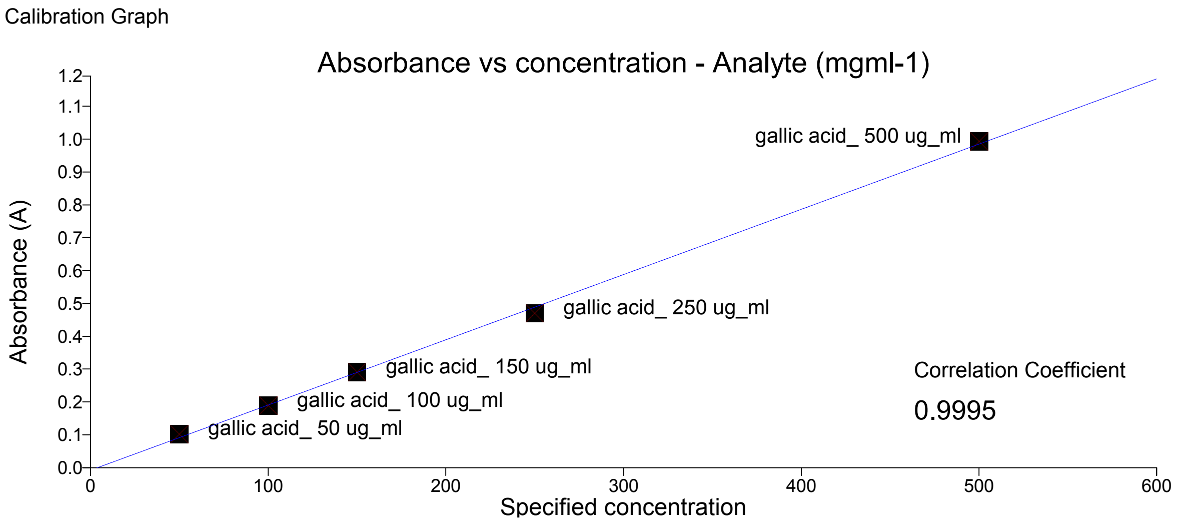

Figure S2. *Correlation of the peak area obtained by UV-visible spectrophotometer and analytical standard concentration of gallic acid for the determination of total phenolic content.*

-

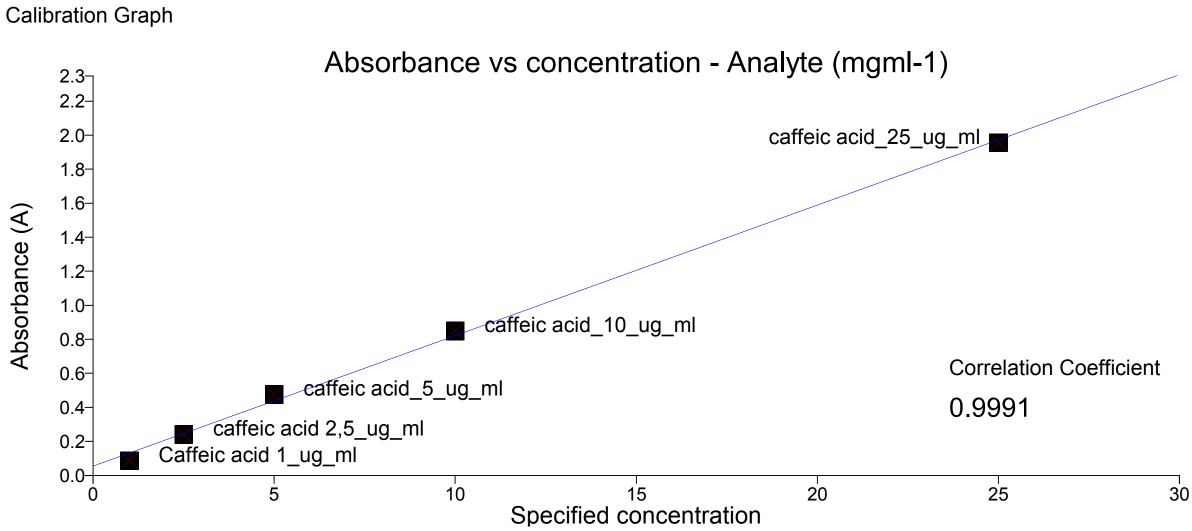

Figure S3. *Correlation of the peak area obtained by UV-visible spectrophotometer and analytical standard concentration of caffeic acid for the determination of hydroxycinnamic acids content.*

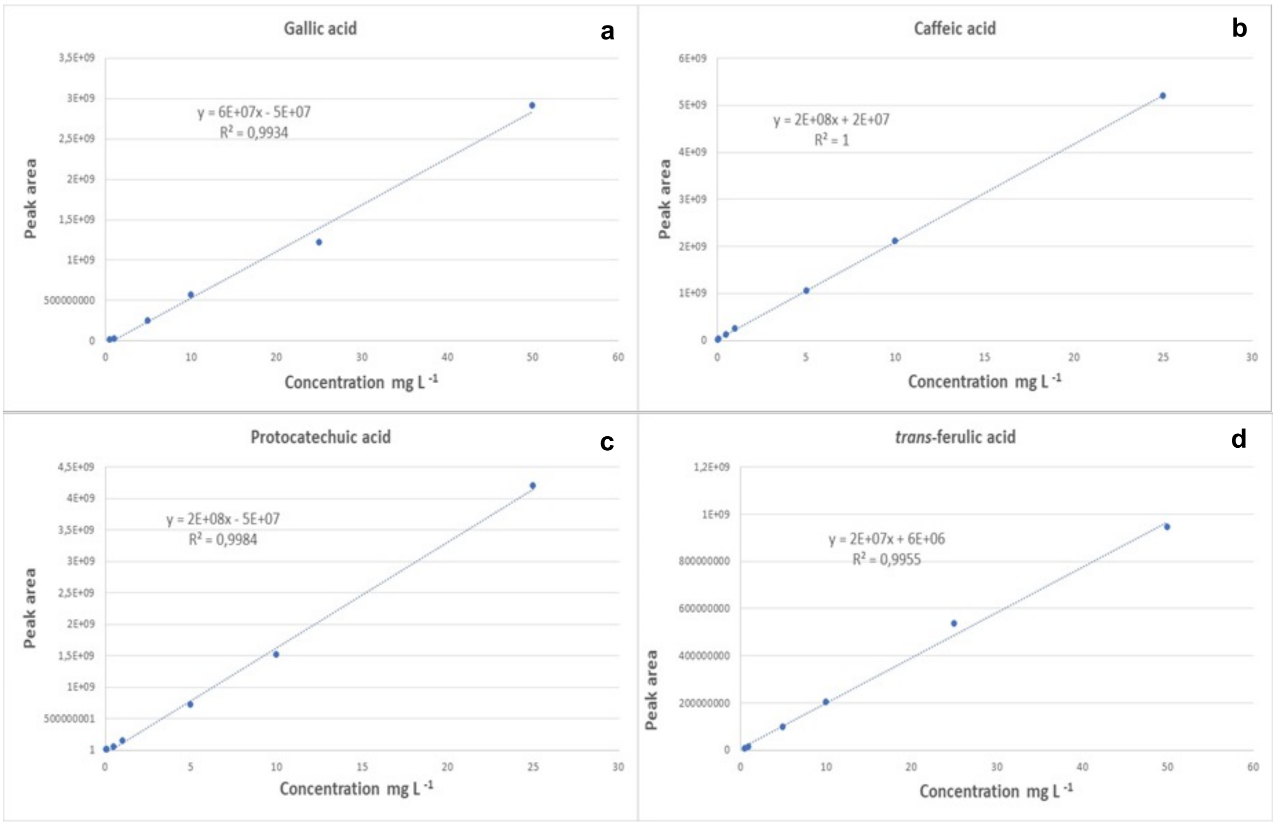

Figure S4. *Correlation of the peak area obtained by UHPLC-ESI-HRMS and analytical standard concentration of gallic acid (a); caffeic acid (b); protocatechuic acid (c); trans-ferulic acid (d)*

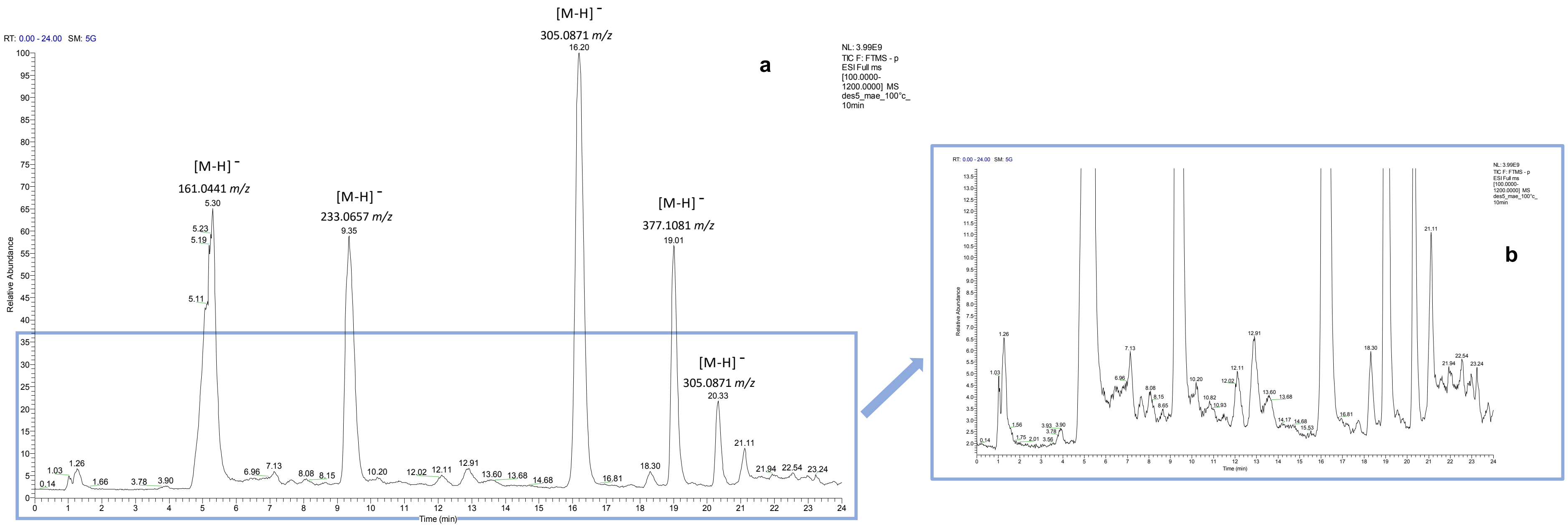

Figure S5. *Full Scan chromatogram obtained by UHPLC-ESI-HRMS for the DES5_MAE_100°C_10 min sample, highlighting the products of a spontaneous polymerization of lactic acid (a) and zoom of sub-spectrum (b)*

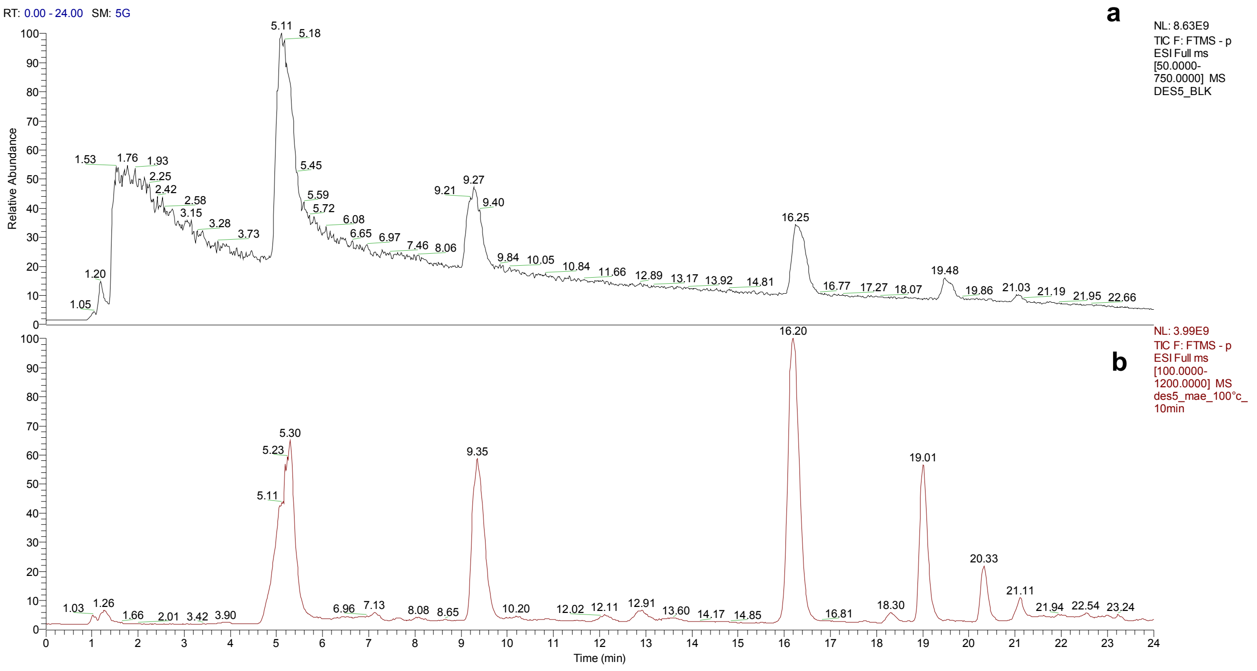

Figure S6. *Comparison between Full Scan chromatograms obtained by UHPLC-ESI-HRMS for the DES5_BLK sample (a) and DES5_MAE_100°C_10 min sample (b).*

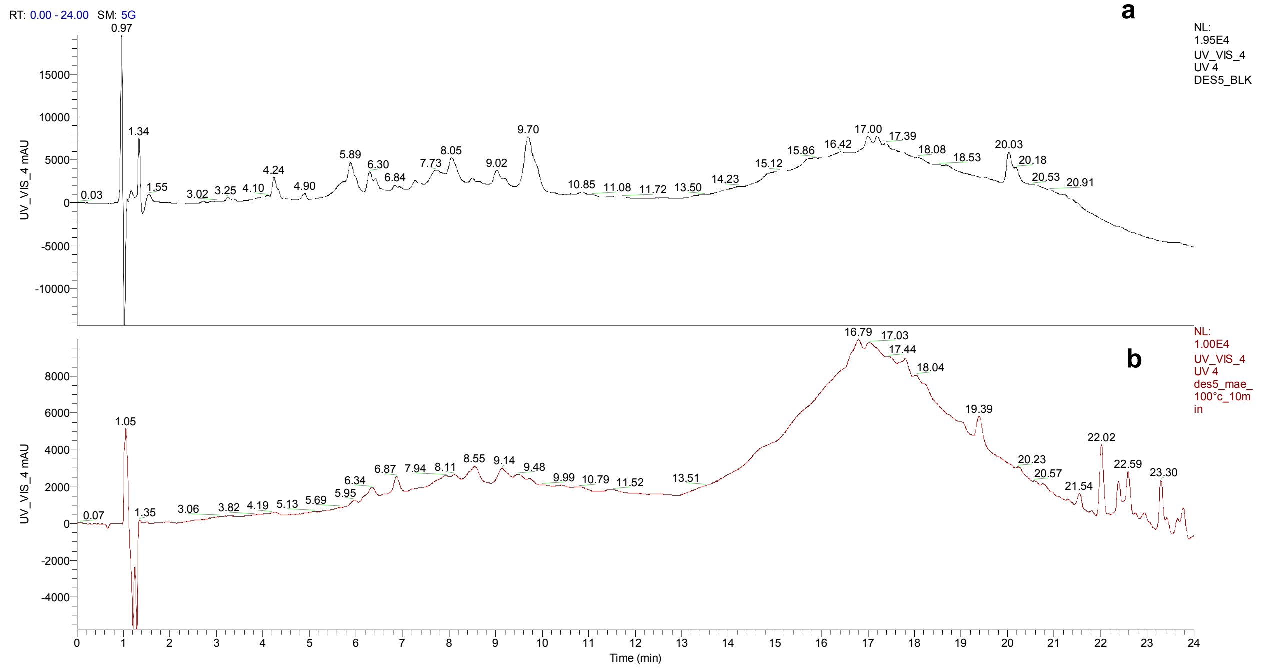

Figure S7. *Comparison between chromatograms obtained by UHPLC-UV/VIS (330nm) for the DES5_BLK sample (a) and DES5_MAE_100°C_10 min sample (b).*

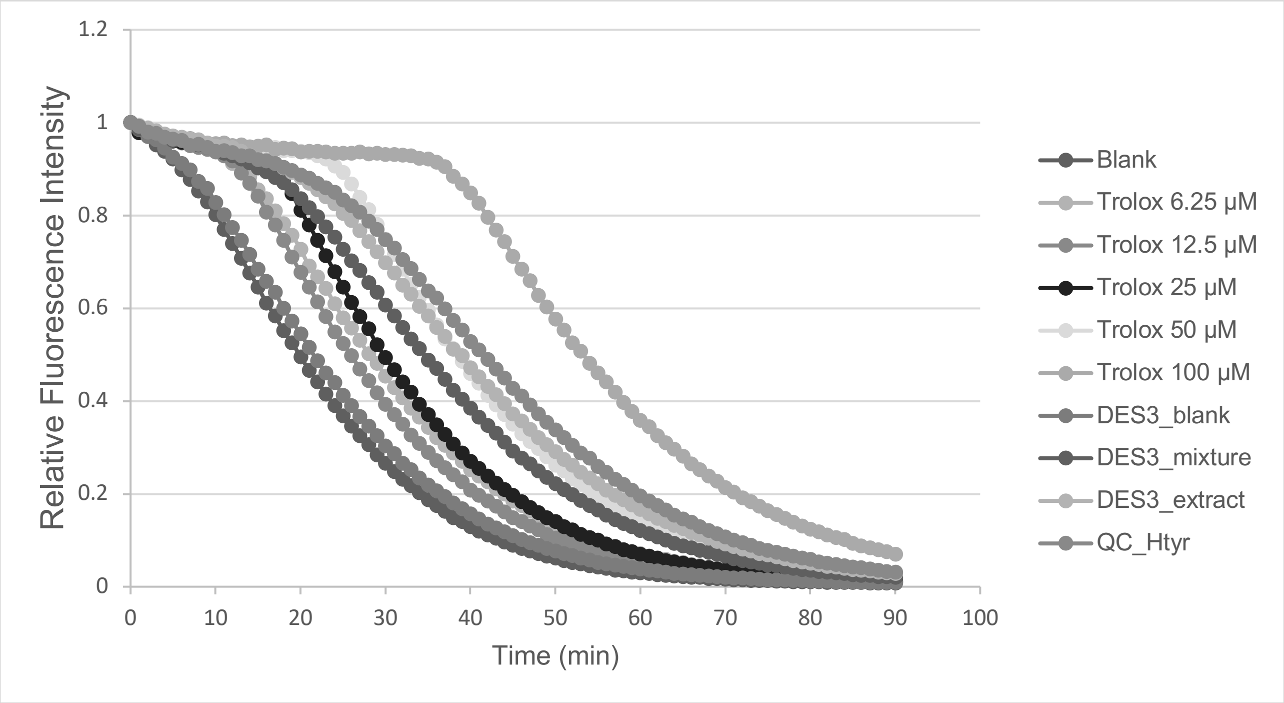

Figure S8. *FL fluorescence decay curve induced by AAPH.*

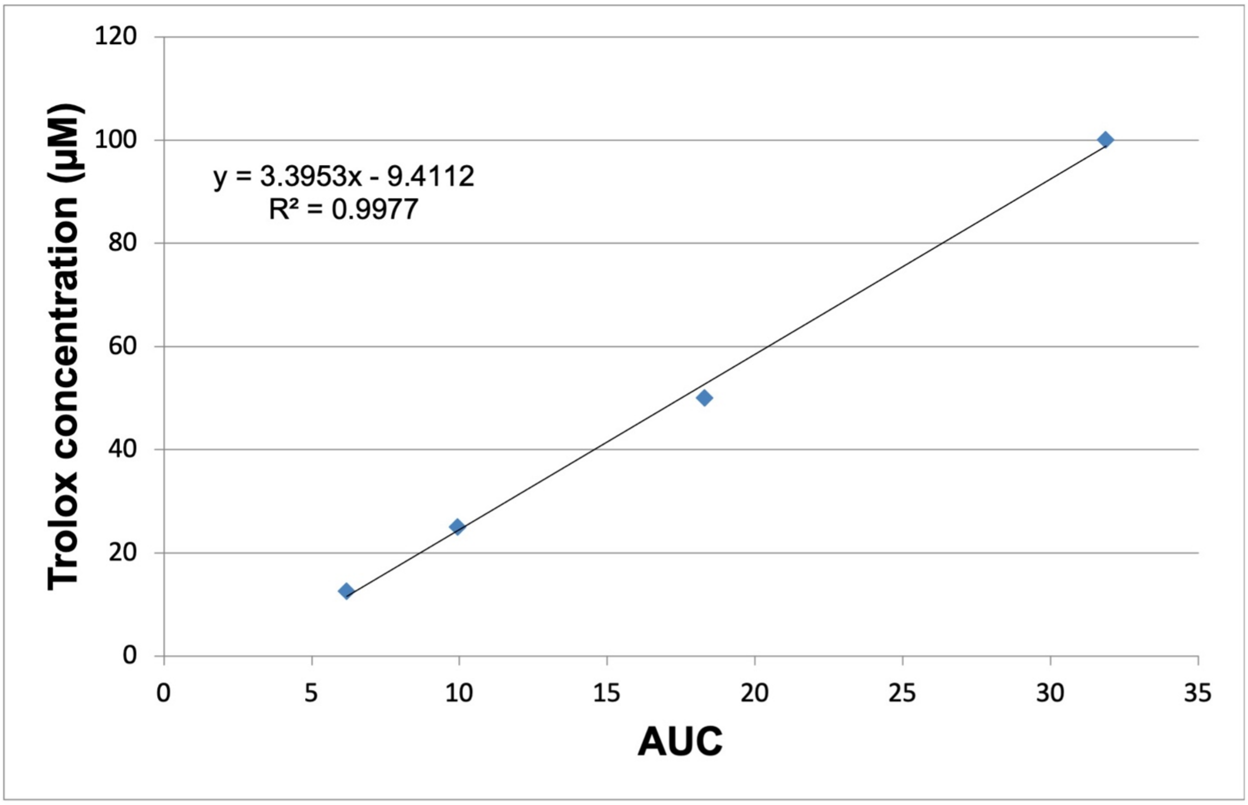

Figure S9. *Linear plot of AUC vs Trolox concentrations.*

Table S1. *Tukye's multiple comparisons test on Table 2*

Compare cell means with others in its column	
Number of families	13
Number of comparisons per column family	45
Alpha	0,05

Turkye's multiple comparisons test	Mean Diff,	95,00% CI of diff,	below treshiold	Summary	Adjusted P Value
TPC					
HA1 vs. HA2	1,830	-0,2863 to 3,946	No	ns	0,1820
HA1 vs. HA3	3,260	1,144 to 5,376	Yes	****	<0,0001
HA1 vs. HA4	4,510	2,394 to 6,626	Yes	****	<0,0001
HA1 vs. HA5	5,170	3,054 to 7,286	Yes	****	<0,0001
HA1 vs. DES1	-15,99	-18,11 to -13,87	Yes	****	<0,0001
HA1 vs. DES2	-8,440	-10,56 to -6,324	Yes	****	<0,0001
HA1 vs. DES3	-39,04	-41,16 to -36,92	Yes	****	<0,0001
HA1 vs. DES4	-12,83	-14,95 to -10,71	Yes	****	<0,0001
HA1 vs. DES5	-29,78	-31,90 to -27,66	Yes	****	<0,0001
HA2 vs. HA3	1,430	-0,6863 to 3,546	No	ns	0,6706
HA2 vs. HA4	2,680	0,5637 to 4,796	Yes	**	0,0026
HA2 vs. HA5	3,340	1,224 to 5,456	Yes	****	<0,0001
HA2 vs. DES1	-17,82	-19,94 to -15,70	Yes	****	<0,0001
HA2 vs. DES2	-10,27	-12,39 to -8,154	Yes	****	<0,0001
HA2 vs. DES3	-40,87	-42,99 to -38,75	Yes	****	<0,0001
HA2 vs. DES4	-14,66	-16,78 to -12,54	Yes	****	<0,0001
HA2 vs. DES5	-31,61	-33,73 to -29,49	Yes	****	<0,0001
HA3 vs. HA4	1,250	-0,8663 to 3,366	No	ns	0,8906
HA3 vs. HA5	1,910	-0,2063 to 4,026	No	ns	0,1296
HA3 vs. DES1	-19,25	-21,37 to -17,13	Yes	****	<0,0001
HA3 vs. DES2	-11,70	-13,82 to -9,584	Yes	****	<0,0001
HA3 vs. DES3	-42,30	-44,42 to -40,18	Yes	****	<0,0001
HA3 vs. DES4	-16,09	-18,21 to -13,97	Yes	****	<0,0001
HA3 vs. DES5	-33,04	-35,16 to -30,92	Yes	****	<0,0001
HA4 vs. HA5	0,6600	-1,456 to 2,776	No	ns	>0,9999
HA4 vs. DES1	-20,50	-22,62 to -18,38	Yes	****	<0,0001
HA4 vs. DES2	-12,95	-15,07 to -10,83	Yes	****	<0,0001
HA4 vs. DES3	-43,55	-45,67 to -41,43	Yes	****	<0,0001
HA4 vs. DES4	-17,34	-19,46 to -15,22	Yes	****	<0,0001
HA4 vs. DES5	-34,29	-36,41 to -32,17	Yes	****	<0,0001
HA5 vs. DES1	-21,16	-23,28 to -19,04	Yes	****	<0,0001
HA5 vs. DES2	-13,61	-15,73 to -11,49	Yes	****	<0,0001
HA5 vs. DES3	-44,21	-46,33 to -42,09	Yes	****	<0,0001
HA5 vs. DES4	-18,00	-20,12 to -15,88	Yes	****	<0,0001
HA5 vs. DES5	-34,95	-37,07 to -32,83	Yes	****	<0,0001
DES1 vs. DES2	7,550	5,434 to 9,666	Yes	****	<0,0001
DES1 vs. DES3	-23,05	-25,17 to -20,93	Yes	****	<0,0001
DES1 vs. DES4	3,160	1,044 to 5,276	Yes	***	0,0002
DES1 vs. DES5	-13,79	-15,91 to -11,67	Yes	****	<0,0001
DES2 vs. DES3	-30,60	-32,72 to -28,48	Yes	****	<0,0001
DES2 vs. DES4	-4,390	-6,506 to -2,274	Yes	****	<0,0001
DES2 vs. DES5	-21,34	-23,46 to -19,22	Yes	****	<0,0001
DES3 vs. DES4	26,21	24,09 to 28,33	Yes	****	<0,0001
DES3 vs. DES5	9,260	7,144 to 11,38	Yes	****	<0,0001
DES4 vs. DES5	-16,95	-19,07 to -14,83	Yes	****	<0,0001
HCA					
HA1 vs. HA2	-0,4500	-2,566 to 1,666	No	ns	>0,9999
HA1 vs. HA3	-0,1100	-2,226 to 2,006	No	ns	>0,9999
HA1 vs. HA4	0,08000	-2,036 to 2,196	No	ns	>0,9999
HA1 vs. HA5	0,3500	-1,766 to 2,466	No	ns	>0,9999
HA1 vs. DES1	-1,970	-4,086 to 0,1463	No	ns	0,0992
HA1 vs. DES2	-0,5900	-2,706 to 1,526	No	ns	>0,9999
HA1 vs. DES3	-3,290	-5,406 to -1,174	Yes	****	<0,0001
HA1 vs. DES4	1,400	-0,7163 to 3,516	No	ns	0,7135
HA1 vs. DES5	-2,610	-4,726 to -0,4937	Yes	**	0,0038
HA2 vs. HA3	0,3400	-1,776 to 2,456	No	ns	>0,9999
HA2 vs. HA4	0,5300	-1,586 to 2,646	No	ns	>0,9999
HA2 vs. HA5	0,8000	-1,316 to 2,916	No	ns	>0,9999
HA2 vs. DES1	-1,520	-3,636 to 0,5963	No	ns	0,5379
HA2 vs. DES2	-0,1400	-2,256 to 1,976	No	ns	>0,9999
HA2 vs. DES3	-2,840	-4,956 to -0,7237	Yes	**	0,0011
HA2 vs. DES4	1,850	-0,2663 to 3,966	No	ns	0,1675

HA2 vs. DES5	-2,160	-4,276 to -0,04373	Yes	*	0,0404
HA3 vs. HA4	0,1900	-1,926 to 2,306	No	ns	>0,9999
HA3 vs. HA5	0,4600	-1,656 to 2,576	No	ns	>0,9999
HA3 vs. DES1	-1,860	-3,976 to 0,2563	No	ns	0,1606
HA3 vs. DES2	-0,4800	-2,596 to 1,636	No	ns	>0,9999
HA3 vs. DES3	-3,180	-5,296 to -1,064	Yes	***	0,0001
HA3 vs. DES4	1,510	-0,6063 to 3,626	No	ns	0,5526
HA3 vs. DES5	-2,500	-4,616 to -0,3837	Yes	**	0,0070
HA4 vs. HA5	0,2700	-1,846 to 2,386	No	ns	>0,9999
HA4 vs. DES1	-2,050	-4,166 to 0,06627	No	ns	0,0686
HA4 vs. DES2	-0,6700	-2,786 to 1,446	No	ns	>0,9999
HA4 vs. DES3	-3,370	-5,486 to -1,254	Yes	****	<0,0001
HA4 vs. DES4	1,320	-0,7963 to 3,436	No	ns	0,8177
HA4 vs. DES5	-2,690	-4,806 to -0,5737	Yes	**	0,0025
HA5 vs. DES1	-2,320	-4,436 to -0,2037	Yes	*	0,0180
HA5 vs. DES2	-0,9400	-3,056 to 1,176	No	ns	0,9985
HA5 vs. DES3	-3,640	-5,756 to -1,524	Yes	****	<0,0001
HA5 vs. DES4	1,050	-1,066 to 3,166	No	ns	0,9889
HA5 vs. DES5	-2,960	-5,076 to -0,8437	Yes	***	0,0005
DES1 vs. DES2	1,380	-0,7363 to 3,496	No	ns	0,7411
DES1 vs. DES3	-1,320	-3,436 to 0,7963	No	ns	0,8177
DES1 vs. DES4	3,370	1,254 to 5,486	Yes	****	<0,0001
DES1 vs. DES5	-0,6400	-2,756 to 1,476	No	ns	>0,9999
DES2 vs. DES3	-2,700	-4,816 to -0,5837	Yes	**	0,0023
DES2 vs. DES4	1,990	-0,1263 to 4,106	No	ns	0,0906
DES2 vs. DES5	-2,020	-4,136 to 0,09627	No	ns	0,0789
DES3 vs. DES4	4,690	2,574 to 6,806	Yes	****	<0,0001
DES3 vs. DES5	0,6800	-1,436 to 2,796	No	ns	>0,9999
DES4 vs. DES5	-4,010	-6,126 to -1,894	Yes	****	<0,0001
TA					
HA1 vs. HA2	0,000	-2,116 to 2,116	No	ns	>0,9999
HA1 vs. HA3	-7,570	-9,686 to -5,454	Yes	****	<0,0001
HA1 vs. HA4	-15,81	-17,93 to -13,69	Yes	****	<0,0001
HA1 vs. HA5	-8,140	-10,26 to -6,024	Yes	****	<0,0001
HA1 vs. DES1	-0,4700	-2,586 to 1,646	No	ns	>0,9999
HA1 vs. DES2	-1,030	-3,146 to 1,086	No	ns	0,9919
HA1 vs. DES3	-2,500	-4,616 to -0,3837	Yes	**	0,0070
HA1 vs. DES4	-0,9100	-3,026 to 1,206	No	ns	0,9992
HA1 vs. DES5	-0,1000	-2,216 to 2,016	No	ns	>0,9999
HA2 vs. HA3	-7,570	-9,686 to -5,454	Yes	****	<0,0001
HA2 vs. HA4	-15,81	-17,93 to -13,69	Yes	****	<0,0001
HA2 vs. HA5	-8,140	-10,26 to -6,024	Yes	****	<0,0001
HA2 vs. DES1	-0,4700	-2,586 to 1,646	No	ns	>0,9999
HA2 vs. DES2	-1,030	-3,146 to 1,086	No	ns	0,9919
HA2 vs. DES3	-2,500	-4,616 to -0,3837	Yes	**	0,0070
HA2 vs. DES4	-0,9100	-3,026 to 1,206	No	ns	0,9992
HA2 vs. DES5	-0,1000	-2,216 to 2,016	No	ns	>0,9999
HA3 vs. HA4	-8,240	-10,36 to -6,124	Yes	****	<0,0001
HA3 vs. HA5	-0,5700	-2,686 to 1,546	No	ns	>0,9999
HA3 vs. DES1	7,100	4,984 to 9,216	Yes	****	<0,0001
HA3 vs. DES2	6,540	4,424 to 8,656	Yes	****	<0,0001
HA3 vs. DES3	5,070	2,954 to 7,186	Yes	****	<0,0001
HA3 vs. DES4	6,660	4,544 to 8,776	Yes	****	<0,0001
HA3 vs. DES5	7,470	5,354 to 9,586	Yes	****	<0,0001
HA4 vs. HA5	7,670	5,554 to 9,786	Yes	****	<0,0001
HA4 vs. DES1	15,34	13,22 to 17,46	Yes	****	<0,0001
HA4 vs. DES2	14,78	12,66 to 16,90	Yes	****	<0,0001
HA4 vs. DES3	13,31	11,19 to 15,43	Yes	****	<0,0001
HA4 vs. DES4	14,90	12,78 to 17,02	Yes	****	<0,0001
HA4 vs. DES5	15,71	13,59 to 17,83	Yes	****	<0,0001
HA5 vs. DES1	7,670	5,554 to 9,786	Yes	****	<0,0001
HA5 vs. DES2	7,110	4,994 to 9,226	Yes	****	<0,0001
HA5 vs. DES3	5,640	3,524 to 7,756	Yes	****	<0,0001
HA5 vs. DES4	7,230	5,114 to 9,346	Yes	****	<0,0001
HA5 vs. DES5	8,040	5,924 to 10,16	Yes	****	<0,0001
DES1 vs. DES2	-0,5600	-2,676 to 1,556	No	ns	>0,9999
DES1 vs. DES3	-2,030	-4,146 to 0,08627	No	ns	0,0753
DES1 vs. DES4	-0,4400	-2,556 to 1,676	No	ns	>0,9999
DES1 vs. DES5	0,3700	-1,746 to 2,486	No	ns	>0,9999
DES2 vs. DES3	-1,470	-3,586 to 0,6463	No	ns	0,6118
DES2 vs. DES4	0,1200	-1,996 to 2,236	No	ns	>0,9999

DES2 vs. DES5	0,9300	-1,186 to 3,046	No	ns	0,9988
DES3 vs. DES4	1,590	-0,5263 to 3,706	No	ns	0,4384
DES3 vs. DES5	2,400	0,2837 to 4,516	Yes	*	0,0119
DES4 vs. DES5	0,8100	-1,306 to 2,926	No	ns	>0,9999

Table S2. *Tukey's multiple comparisons test on Table 3 (against DES3)*

Compare cell means with others in its column					
Number of families	11				
Number of comparisons per column family	28				
Alpha	0,05				
Tukey's multiple comparisons test	Mean Diff,	95,00% CI of diff,	below tresh	Summary	Adjusted P Value
Group A					
DES3 MAE 65 10 vs. DES3 MAE 65 30	11,67	8,251 to 15,09	Yes	****	<0,0001
DES3 MAE 65 10 vs. DES3 MAE 65 45	3,740	0,3212 to 7,159	Yes	*	0,0230
DES3 MAE 65 10 vs. DES3 MAE 100 10	-9,370	-12,79 to -5,951	Yes	****	<0,0001
DES3 MAE 65 10 vs. DES3 UAE 10	-0,8500	-4,269 to 2,569	No	ns	0,9930
DES3 MAE 65 10 vs. DES3 UAE 30	0,3500	-3,069 to 3,769	No	ns	>0,9999
DES3 MAE 65 10 vs. DES3 UAE 45	-1,590	-5,009 to 1,829	No	ns	0,8173
DES3 MAE 65 10 vs. DES3	-26,34	-29,76 to -22,92	Yes	****	<0,0001
DES3 MAE 65 30 vs. DES3 MAE 65 45	-7,930	-11,35 to -4,511	Yes	****	<0,0001
DES3 MAE 65 30 vs. DES3 MAE 100 10	-21,04	-24,46 to -17,62	Yes	****	<0,0001
DES3 MAE 65 30 vs. DES3 UAE 10	-12,52	-15,94 to -9,101	Yes	****	<0,0001
DES3 MAE 65 30 vs. DES3 UAE 30	-11,32	-14,74 to -7,901	Yes	****	<0,0001
DES3 MAE 65 30 vs. DES3 UAE 45	-13,26	-16,68 to -9,841	Yes	****	<0,0001
DES3 MAE 65 30 vs. DES3	-38,01	-41,43 to -34,59	Yes	****	<0,0001
DES3 MAE 65 45 vs. DES3 MAE 100 10	-13,11	-16,53 to -9,691	Yes	****	<0,0001
DES3 MAE 65 45 vs. DES3 UAE 10	-4,590	-8,009 to -1,171	Yes	**	0,0023
DES3 MAE 65 45 vs. DES3 UAE 30	-3,390	-6,809 to 0,02880	No	ns	0,0534
DES3 MAE 65 45 vs. DES3 UAE 45	-5,330	-8,749 to -1,911	Yes	***	0,0003
DES3 MAE 65 45 vs. DES3	-30,08	-33,50 to -26,66	Yes	****	<0,0001
DES3 MAE 100 10 vs. DES3 UAE 10	8,520	5,101 to 11,94	Yes	****	<0,0001
DES3 MAE 100 10 vs. DES3 UAE 30	9,720	6,301 to 13,14	Yes	****	<0,0001
DES3 MAE 100 10 vs. DES3 UAE 45	7,780	4,361 to 11,20	Yes	****	<0,0001
DES3 MAE 100 10 vs. DES3	-16,97	-20,39 to -13,55	Yes	****	<0,0001
DES3 UAE 10 vs. DES3 UAE 30	1,200	-2,219 to 4,619	No	ns	0,9510
DES3 UAE 10 vs. DES3 UAE 45	-0,7400	-4,159 to 2,679	No	ns	0,9970
DES3 UAE 10 vs. DES3	-25,49	-28,91 to -22,07	Yes	****	<0,0001
DES3 UAE 30 vs. DES3 UAE 45	-1,940	-5,359 to 1,479	No	ns	0,6245
DES3 UAE 30 vs. DES3	-26,69	-30,11 to -23,27	Yes	****	<0,0001
DES3 UAE 45 vs. DES3	-24,75	-28,17 to -21,33	Yes	****	<0,0001
Group B					
DES3 MAE 65 10 vs. DES3 MAE 65 30	1,540	-1,879 to 4,959	No	ns	0,8402
DES3 MAE 65 10 vs. DES3 MAE 65 45	0,6700	-2,749 to 4,089	No	ns	0,9984
DES3 MAE 65 10 vs. DES3 MAE 100 10	-0,2600	-3,679 to 3,159	No	ns	>0,9999
DES3 MAE 65 10 vs. DES3 UAE 10	1,400	-2,019 to 4,819	No	ns	0,8954
DES3 MAE 65 10 vs. DES3 UAE 30	2,790	-0,6288 to 6,209	No	ns	0,1865
DES3 MAE 65 10 vs. DES3 UAE 45	1,720	-1,699 to 5,139	No	ns	0,7514
DES3 MAE 65 10 vs. DES3	-0,4700	-3,889 to 2,949	No	ns	0,9998
DES3 MAE 65 30 vs. DES3 MAE 65 45	-0,8700	-4,289 to 2,549	No	ns	0,9920
DES3 MAE 65 30 vs. DES3 MAE 100 10	-1,800	-5,219 to 1,619	No	ns	0,7070
DES3 MAE 65 30 vs. DES3 UAE 10	-0,1400	-3,559 to 3,279	No	ns	>0,9999
DES3 MAE 65 30 vs. DES3 UAE 30	1,250	-2,169 to 4,669	No	ns	0,9396
DES3 MAE 65 30 vs. DES3 UAE 45	0,1800	-3,239 to 3,599	No	ns	>0,9999
DES3 MAE 65 30 vs. DES3	-2,010	-5,429 to 1,409	No	ns	0,5821
DES3 MAE 65 45 vs. DES3 MAE 100 10	-0,9300	-4,349 to 2,489	No	ns	0,9881
DES3 MAE 65 45 vs. DES3 UAE 10	0,7300	-2,689 to 4,149	No	ns	0,9973
DES3 MAE 65 45 vs. DES3 UAE 30	2,120	-1,299 to 5,539	No	ns	0,5154
DES3 MAE 65 45 vs. DES3 UAE 45	1,050	-2,369 to 4,469	No	ns	0,9761
DES3 MAE 65 45 vs. DES3	-1,140	-4,559 to 2,279	No	ns	0,9626
DES3 MAE 100 10 vs. DES3 UAE 10	1,660	-1,759 to 5,079	No	ns	0,7830
DES3 MAE 100 10 vs. DES3 UAE 30	3,050	-0,3688 to 6,469	No	ns	0,1123
DES3 MAE 100 10 vs. DES3 UAE 45	1,980	-1,439 to 5,399	No	ns	0,6003
DES3 MAE 100 10 vs. DES3	-0,2100	-3,629 to 3,209	No	ns	>0,9999
DES3 UAE 10 vs. DES3 UAE 30	1,390	-2,029 to 4,809	No	ns	0,8988
DES3 UAE 10 vs. DES3 UAE 45	0,3200	-3,099 to 3,739	No	ns	>0,9999
DES3 UAE 10 vs. DES3	-1,870	-5,289 to 1,549	No	ns	0,6663
DES3 UAE 30 vs. DES3 UAE 45	-1,070	-4,489 to 2,349	No	ns	0,9735
DES3 UAE 30 vs. DES3	-3,260	-6,679 to 0,1588	No	ns	0,0717

DES3 UAE 45 vs. DES3	-2,190	-5,609 to 1,229	No	ns	0,4737
Group C					
DES3 MAE 65 10 vs. DES3 MAE 65 30	1,410	-2,009 to 4,829	No	ns	0,8919
DES3 MAE 65 10 vs. DES3 MAE 65 45	1,280	-2,139 to 4,699	No	ns	0,9320
DES3 MAE 65 10 vs. DES3 MAE 100 10	3,450	0,03120 to 6,869	Yes	*	0,0465
DES3 MAE 65 10 vs. DES3 UAE 10	3,220	-0,1988 to 6,639	No	ns	0,0783
DES3 MAE 65 10 vs. DES3 UAE 30	3,730	0,3112 to 7,149	Yes	*	0,0236
DES3 MAE 65 10 vs. DES3 UAE 45	3,730	0,3112 to 7,149	Yes	*	0,0236
DES3 MAE 65 10 vs. DES3	1,050	-2,369 to 4,469	No	ns	0,9761
DES3 MAE 65 30 vs. DES3 MAE 65 45	-0,1300	-3,549 to 3,289	No	ns	>0,9999
DES3 MAE 65 30 vs. DES3 MAE 100 10	2,040	-1,379 to 5,459	No	ns	0,5639
DES3 MAE 65 30 vs. DES3 UAE 10	1,810	-1,609 to 5,229	No	ns	0,7013
DES3 MAE 65 30 vs. DES3 UAE 30	2,320	-1,099 to 5,739	No	ns	0,3993
DES3 MAE 65 30 vs. DES3 UAE 45	2,320	-1,099 to 5,739	No	ns	0,3993
DES3 MAE 65 30 vs. DES3	-0,3600	-3,779 to 3,059	No	ns	>0,9999
DES3 MAE 65 45 vs. DES3 MAE 100 10	2,170	-1,249 to 5,589	No	ns	0,4855
DES3 MAE 65 45 vs. DES3 UAE 10	1,940	-1,479 to 5,359	No	ns	0,6245
DES3 MAE 65 45 vs. DES3 UAE 30	2,450	-0,9688 to 5,869	No	ns	0,3306
DES3 MAE 65 45 vs. DES3 UAE 45	2,450	-0,9688 to 5,869	No	ns	0,3306
DES3 MAE 65 45 vs. DES3	-0,2300	-3,649 to 3,189	No	ns	>0,9999
DES3 MAE 100 10 vs. DES3 UAE 10	-0,2300	-3,649 to 3,189	No	ns	>0,9999
DES3 MAE 100 10 vs. DES3 UAE 30	0,2800	-3,139 to 3,699	No	ns	>0,9999
DES3 MAE 100 10 vs. DES3 UAE 45	0,2800	-3,139 to 3,699	No	ns	>0,9999
DES3 MAE 100 10 vs. DES3	-2,400	-5,819 to 1,019	No	ns	0,3562
DES3 UAE 10 vs. DES3 UAE 30	0,5100	-2,909 to 3,929	No	ns	0,9997
DES3 UAE 10 vs. DES3 UAE 45	0,5100	-2,909 to 3,929	No	ns	0,9997
DES3 UAE 10 vs. DES3	-2,170	-5,589 to 1,249	No	ns	0,4855
DES3 UAE 30 vs. DES3 UAE 45	0,000	-3,419 to 3,419	No	ns	>0,9999
DES3 UAE 30 vs. DES3	-2,680	-6,099 to 0,7388	No	ns	0,2271
DES3 UAE 45 vs. DES3	-2,680	-6,099 to 0,7388	No	ns	0,2271

Table S3. *Tukey's multiple comparisons test on Table 3 (against DES5)*

Compare cell means with others in its column					
Number of families	11				
Number of comparisons per column family	28				
Alpha	0,05				
Tukey's multiple comparisons test	Mean Diff,	95,00% CI of diff,	Below tresh.	Summary	Adjusted P Value
DES5 MAE 65 10					
Group A vs. Group B	42,68	37,16 to 48,20	Yes	****	<0,0001
Group A vs. Group C	46,93	41,41 to 52,45	Yes	****	<0,0001
Group B vs. Group C	4,250	-1,270 to 9,770	No	ns	0,1608
DES5 MAE 65 30					
Group A vs. Group B	23,36	17,84 to 28,88	Yes	****	<0,0001
Group A vs. Group C	26,63	21,11 to 32,15	Yes	****	<0,0001
Group B vs. Group C	3,270	-2,250 to 8,790	No	ns	0,3324
DES5 MAE 65 45					
Group A vs. Group B	38,95	33,43 to 44,47	Yes	****	<0,0001
Group A vs. Group C	43,66	38,14 to 49,18	Yes	****	<0,0001
Group B vs. Group C	4,710	-0,8097 to 10,23	No	ns	0,1083
DES5 MAE 100 10					
Group A vs. Group B	22,39	16,87 to 27,91	Yes	****	<0,0001
Group A vs. Group C	32,20	26,68 to 37,72	Yes	****	<0,0001
Group B vs. Group C	9,810	4,290 to 15,33	Yes	***	0,0002
DES5 UAE 10					
Group A vs. Group B	35,12	29,60 to 40,64	Yes	****	<0,0001
Group A vs. Group C	38,78	33,26 to 44,30	Yes	****	<0,0001
Group B vs. Group C	3,660	-1,860 to 9,180	No	ns	0,2539
DES5 UAE 30					
Group A vs. Group B	32,17	26,65 to 37,69	Yes	****	<0,0001
Group A vs. Group C	35,46	29,94 to 40,98	Yes	****	<0,0001
Group B vs. Group C	3,290	-2,230 to 8,810	No	ns	0,3281
DES5 UAE 45					
Group A vs. Group B	37,05	31,53 to 42,57	Yes	****	<0,0001
Group A vs. Group C	42,29	36,77 to 47,81	Yes	****	<0,0001
Group B vs. Group C	5,240	-0,2797 to 10,76	No	ns	0,0660
DES5					
Group A vs. Group B	47,98	42,46 to 53,50	Yes	****	<0,0001
Group A vs. Group C	53,07	47,55 to 58,59	Yes	****	<0,0001
Group B vs. Group C	5,090	-0,4297 to 10,61	No	ns	0,0762

Group A					
DES5 MAE 65 10 vs. DES5 MAE 65 30	20,31	13,08 to 27,54	Yes	****	<0,0001
DES5 MAE 65 10 vs. DES5 MAE 65 45	3,220	-4,011 to 10,45	No	ns	0,8478
DES5 MAE 65 10 vs. DES5 MAE 100 10	14,77	7,539 to 22,00	Yes	****	<0,0001
DES5 MAE 65 10 vs. DES5 UAE 10	8,190	0,9590 to 15,42	Yes	*	0,0164
DES5 MAE 65 10 vs. DES5 UAE 30	11,51	4,279 to 18,74	Yes	***	0,0002
DES5 MAE 65 10 vs. DES5 UAE 45	4,680	-2,551 to 11,91	No	ns	0,4604
DES5 MAE 65 10 vs. DES5	-6,380	-13,61 to 0,8510	No	ns	0,1202
DES5 MAE 65 30 vs. DES5 MAE 65 45	-17,09	-24,32 to -9,859	Yes	****	<0,0001
DES5 MAE 65 30 vs. DES5 MAE 100 10	-5,540	-12,77 to 1,691	No	ns	0,2520
DES5 MAE 65 30 vs. DES5 UAE 10	-12,12	-19,35 to -4,889	Yes	****	<0,0001
DES5 MAE 65 30 vs. DES5 UAE 30	-8,800	-16,03 to -1,569	Yes	**	0,0076
DES5 MAE 65 30 vs. DES5 UAE 45	-15,63	-22,86 to -8,399	Yes	****	<0,0001
DES5 MAE 65 30 vs. DES5	-26,69	-33,92 to -19,46	Yes	****	<0,0001
DES5 MAE 65 45 vs. DES5 MAE 100 10	11,55	4,319 to 18,78	Yes	***	0,0002
DES5 MAE 65 45 vs. DES5 UAE 10	4,970	-2,261 to 12,20	No	ns	0,3829
DES5 MAE 65 45 vs. DES5 UAE 30	8,290	1,059 to 15,52	Yes	*	0,0145
DES5 MAE 65 45 vs. DES5 UAE 45	1,460	-5,771 to 8,691	No	ns	0,9981
DES5 MAE 65 45 vs. DES5	-9,600	-16,83 to -2,369	Yes	**	0,0027
DES5 MAE 100 10 vs. DES5 UAE 10	-6,580	-13,81 to 0,6510	No	ns	0,0989
DES5 MAE 100 10 vs. DES5 UAE 30	-3,260	-10,49 to 3,971	No	ns	0,8396
DES5 MAE 100 10 vs. DES5 UAE 45	-10,09	-17,32 to -2,859	Yes	**	0,0014
DES5 MAE 100 10 vs. DES5	-21,15	-28,38 to -13,92	Yes	****	<0,0001
DES5 UAE 10 vs. DES5 UAE 30	3,320	-3,911 to 10,55	No	ns	0,8268
DES5 UAE 10 vs. DES5 UAE 45	-3,510	-10,74 to 3,721	No	ns	0,7832
DES5 UAE 10 vs. DES5	-14,57	-21,80 to -7,339	Yes	****	<0,0001
DES5 UAE 30 vs. DES5 UAE 45	-6,830	-14,06 to 0,4010	No	ns	0,0767
DES5 UAE 30 vs. DES5	-17,89	-25,12 to -10,66	Yes	****	<0,0001
DES5 UAE 45 vs. DES5	-11,06	-18,29 to -3,829	Yes	***	0,0003
Group B					
DES5 MAE 65 10 vs. DES5 MAE 65 30	0,9900	-6,241 to 8,221	No	ns	0,9998
DES5 MAE 65 10 vs. DES5 MAE 65 45	-0,5100	-7,741 to 6,721	No	ns	>0,9999
DES5 MAE 65 10 vs. DES5 MAE 100 10	-5,520	-12,75 to 1,711	No	ns	0,2561
DES5 MAE 65 10 vs. DES5 UAE 10	0,6300	-6,601 to 7,861	No	ns	>0,9999
DES5 MAE 65 10 vs. DES5 UAE 30	1,000	-6,231 to 8,231	No	ns	0,9998
DES5 MAE 65 10 vs. DES5 UAE 45	-0,9500	-8,181 to 6,281	No	ns	0,9999
DES5 MAE 65 10 vs. DES5	-1,080	-8,311 to 6,151	No	ns	0,9997
DES5 MAE 65 30 vs. DES5 MAE 65 45	-1,500	-8,731 to 5,731	No	ns	0,9977
DES5 MAE 65 30 vs. DES5 MAE 100 10	-6,510	-13,74 to 0,7210	No	ns	0,1060
DES5 MAE 65 30 vs. DES5 UAE 10	-0,3600	-7,591 to 6,871	No	ns	>0,9999
DES5 MAE 65 30 vs. DES5 UAE 30	0,01000	-7,221 to 7,241	No	ns	>0,9999
DES5 MAE 65 30 vs. DES5 UAE 45	-1,940	-9,171 to 5,291	No	ns	0,9890
DES5 MAE 65 30 vs. DES5	-2,070	-9,301 to 5,161	No	ns	0,9840
DES5 MAE 65 45 vs. DES5 MAE 100 10	-5,010	-12,24 to 2,221	No	ns	0,3728
DES5 MAE 65 45 vs. DES5 UAE 10	1,140	-6,091 to 8,371	No	ns	0,9996
DES5 MAE 65 45 vs. DES5 UAE 30	1,510	-5,721 to 8,741	No	ns	0,9976
DES5 MAE 65 45 vs. DES5 UAE 45	-0,4400	-7,671 to 6,791	No	ns	>0,9999
DES5 MAE 65 45 vs. DES5	-0,5700	-7,801 to 6,661	No	ns	>0,9999
DES5 MAE 100 10 vs. DES5 UAE 10	6,150	-1,081 to 13,38	No	ns	0,1493
DES5 MAE 100 10 vs. DES5 UAE 30	6,520	-0,7110 to 13,75	No	ns	0,1049
DES5 MAE 100 10 vs. DES5 UAE 45	4,570	-2,661 to 11,80	No	ns	0,4911
DES5 MAE 100 10 vs. DES5	4,440	-2,791 to 11,67	No	ns	0,5279
DES5 UAE 10 vs. DES5 UAE 30	0,3700	-6,861 to 7,601	No	ns	>0,9999
DES5 UAE 10 vs. DES5 UAE 45	-1,580	-8,811 to 5,651	No	ns	0,9968
DES5 UAE 10 vs. DES5	-1,710	-8,941 to 5,521	No	ns	0,9948
DES5 UAE 30 vs. DES5 UAE 45	-1,950	-9,181 to 5,281	No	ns	0,9887
DES5 UAE 30 vs. DES5	-2,080	-9,311 to 5,151	No	ns	0,9835
DES5 UAE 45 vs. DES5	-0,1300	-7,361 to 7,101	No	ns	>0,9999
Group C					
DES5 MAE 65 10 vs. DES5 MAE 65 30	0,01000	-7,221 to 7,241	No	ns	>0,9999
DES5 MAE 65 10 vs. DES5 MAE 65 45	-0,05000	-7,281 to 7,181	No	ns	>0,9999
DES5 MAE 65 10 vs. DES5 MAE 100 10	0,04000	-7,191 to 7,271	No	ns	>0,9999
DES5 MAE 65 10 vs. DES5 UAE 10	0,04000	-7,191 to 7,271	No	ns	>0,9999
DES5 MAE 65 10 vs. DES5 UAE 30	0,04000	-7,191 to 7,271	No	ns	>0,9999
DES5 MAE 65 10 vs. DES5 UAE 45	0,04000	-7,191 to 7,271	No	ns	>0,9999
DES5 MAE 65 10 vs. DES5	-0,2400	-7,471 to 6,991	No	ns	>0,9999
DES5 MAE 65 30 vs. DES5 MAE 65 45	-0,06000	-7,291 to 7,171	No	ns	>0,9999
DES5 MAE 65 30 vs. DES5 MAE 100 10	0,03000	-7,201 to 7,261	No	ns	>0,9999
DES5 MAE 65 30 vs. DES5 UAE 10	0,03000	-7,201 to 7,261	No	ns	>0,9999
DES5 MAE 65 30 vs. DES5 UAE 30	0,03000	-7,201 to 7,261	No	ns	>0,9999
DES5 MAE 65 30 vs. DES5 UAE 45	0,03000	-7,201 to 7,261	No	ns	>0,9999

DES5 MAE 65 30 vs. DES5	-0,2500	-7,481 to 6,981	No	ns	>0,9999
DES5 MAE 65 45 vs. DES5 MAE 100 10	0,09000	-7,141 to 7,321	No	ns	>0,9999
DES5 MAE 65 45 vs. DES5 UAE 10	0,09000	-7,141 to 7,321	No	ns	>0,9999
DES5 MAE 65 45 vs. DES5 UAE 30	0,09000	-7,141 to 7,321	No	ns	>0,9999
DES5 MAE 65 45 vs. DES5 UAE 45	0,09000	-7,141 to 7,321	No	ns	>0,9999
DES5 MAE 65 45 vs. DES5	-0,1900	-7,421 to 7,041	No	ns	>0,9999
DES5 MAE 100 10 vs. DES5 UAE 10	0,000	-7,231 to 7,231	No	ns	>0,9999
DES5 MAE 100 10 vs. DES5 UAE 30	0,000	-7,231 to 7,231	No	ns	>0,9999
DES5 MAE 100 10 vs. DES5 UAE 45	0,000	-7,231 to 7,231	No	ns	>0,9999
DES5 MAE 100 10 vs. DES5	-0,2800	-7,511 to 6,951	No	ns	>0,9999
DES5 UAE 10 vs. DES5 UAE 30	0,000	-7,231 to 7,231	No	ns	>0,9999
DES5 UAE 10 vs. DES5 UAE 45	0,000	-7,231 to 7,231	No	ns	>0,9999
DES5 UAE 10 vs. DES5	-0,2800	-7,511 to 6,951	No	ns	>0,9999
DES5 UAE 30 vs. DES5 UAE 45	0,000	-7,231 to 7,231	No	ns	>0,9999
DES5 UAE 30 vs. DES5	-0,2800	-7,511 to 6,951	No	ns	>0,9999
DES5 UAE 45 vs. DES5	-0,2800	-7,511 to 6,951	No	ns	>0,9999

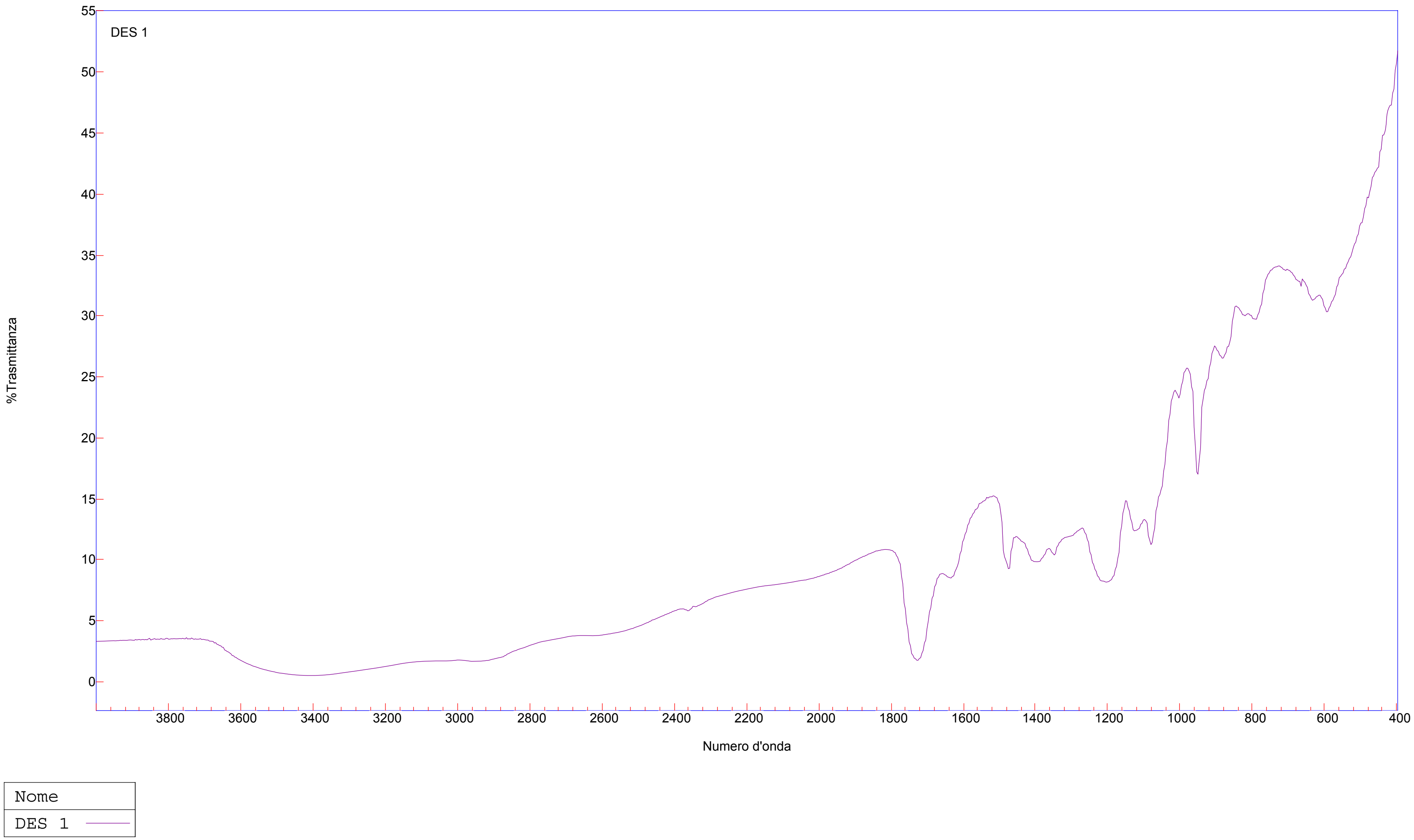

Figure S10. *FT-IR spectrum of DES1.*

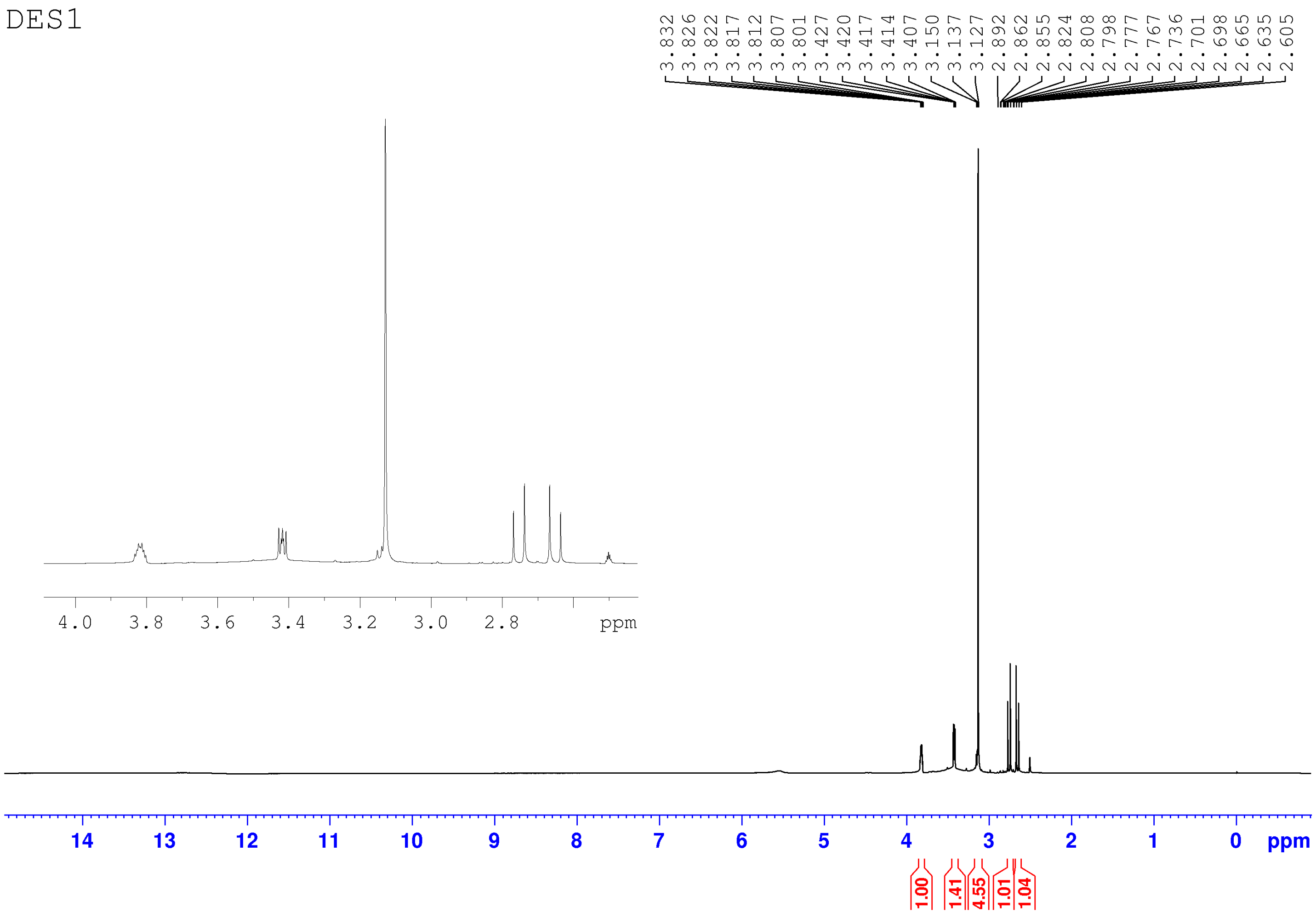

Figure S11. *¹H-NMR spectrum of DES1.*

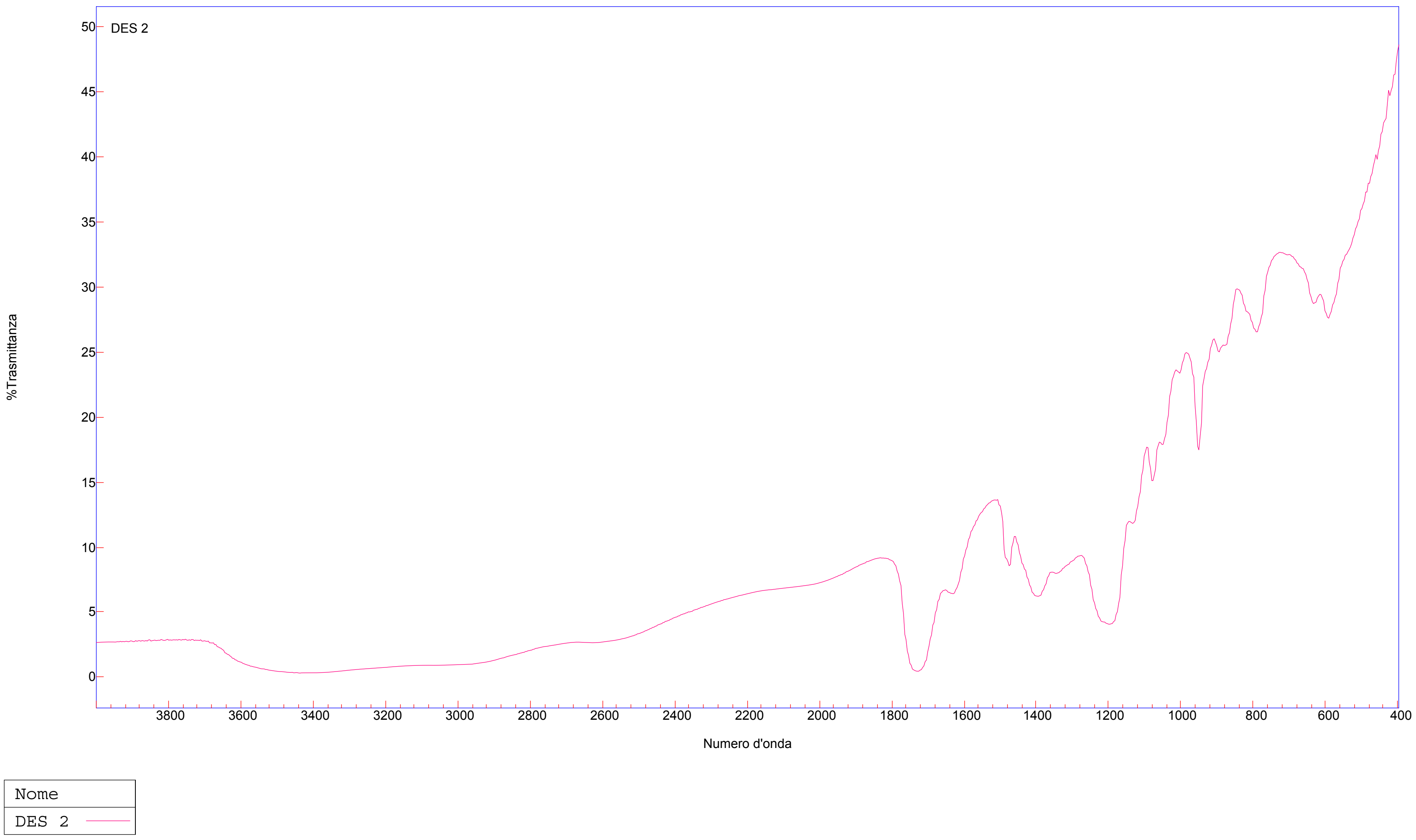

Figure S12. *FT-IR spectrum of DES2.*

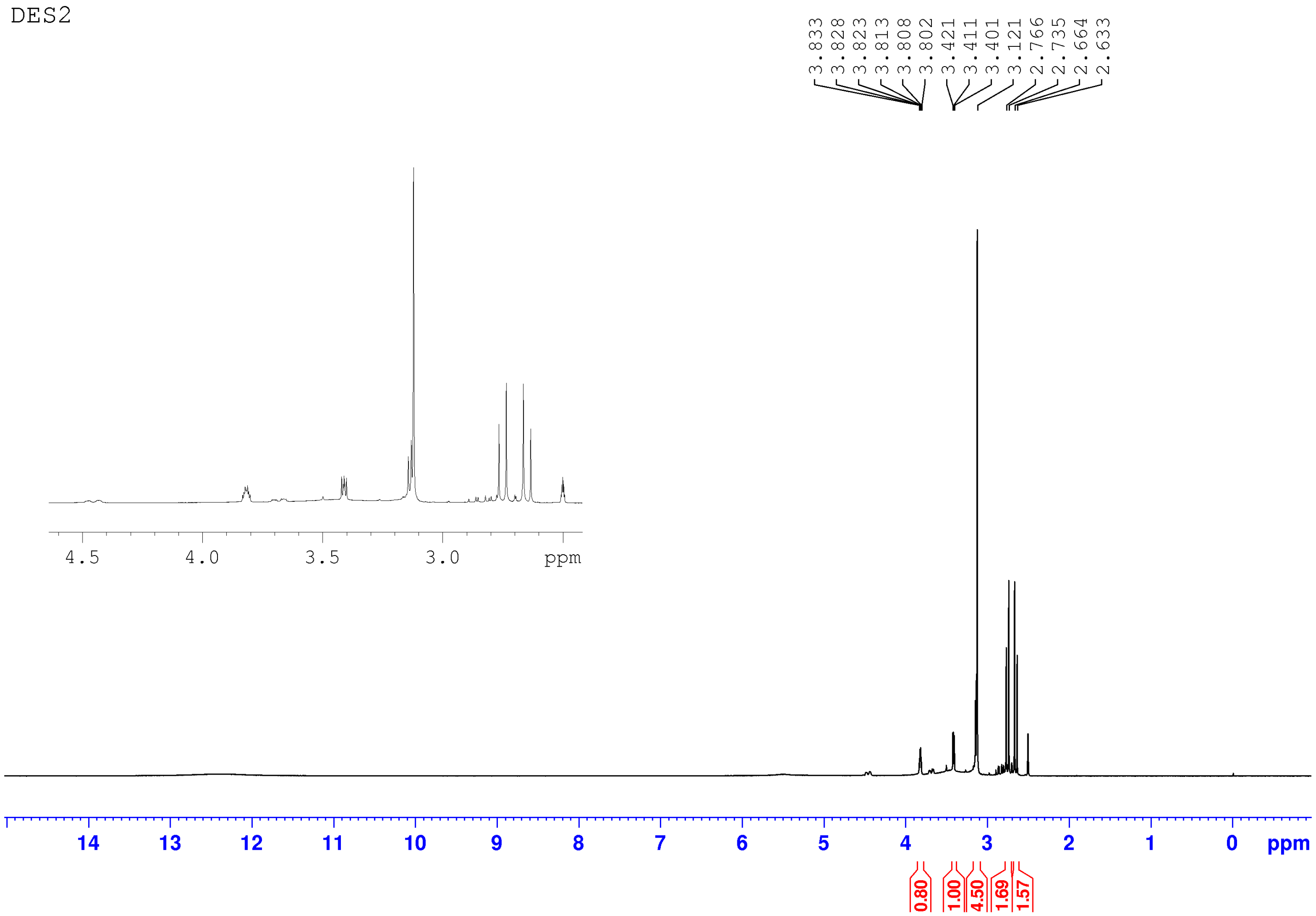

Figure S13. *¹H-NMR spectrum of DES2.*

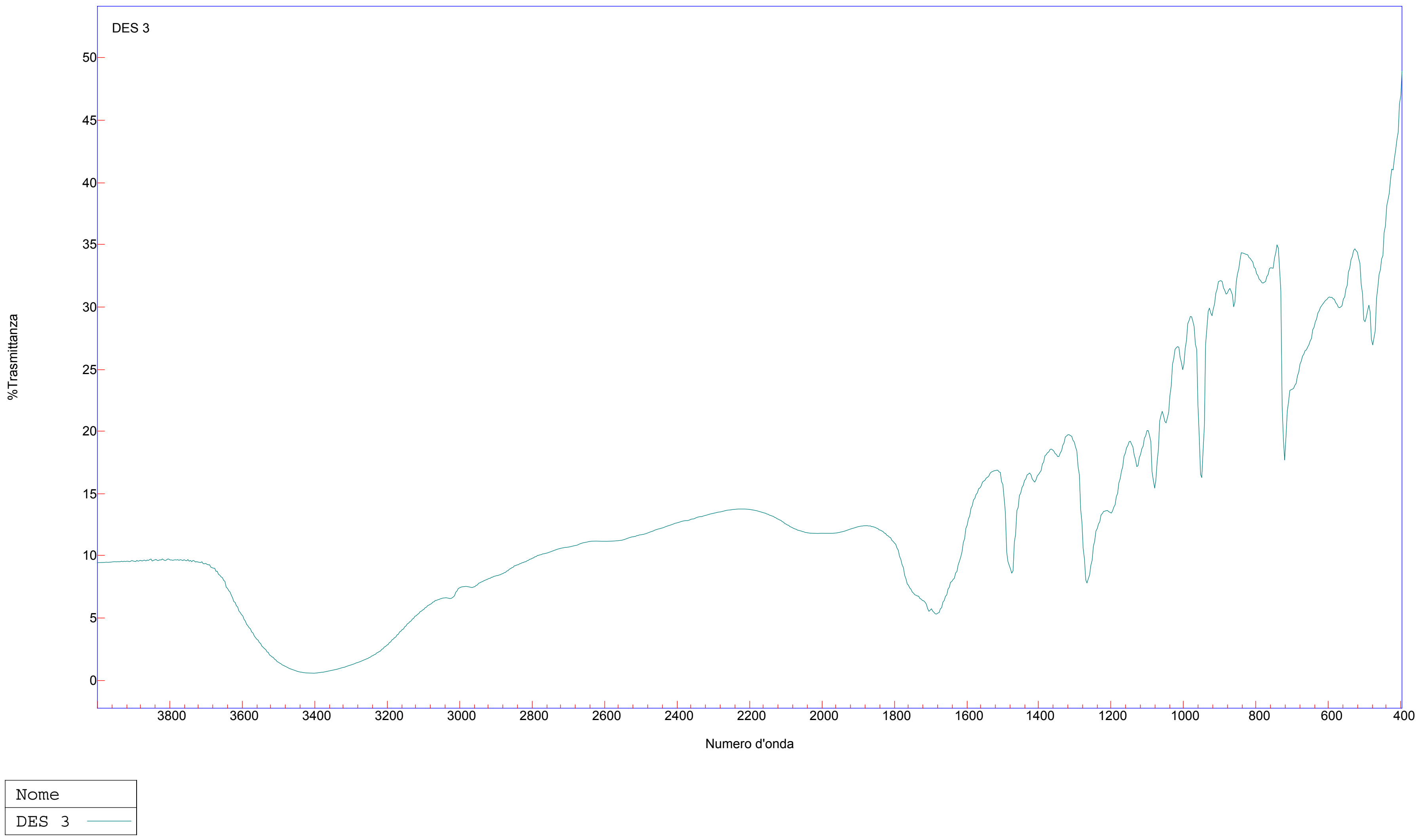

Figure S14. *FT-IR spectrum of DES3.*

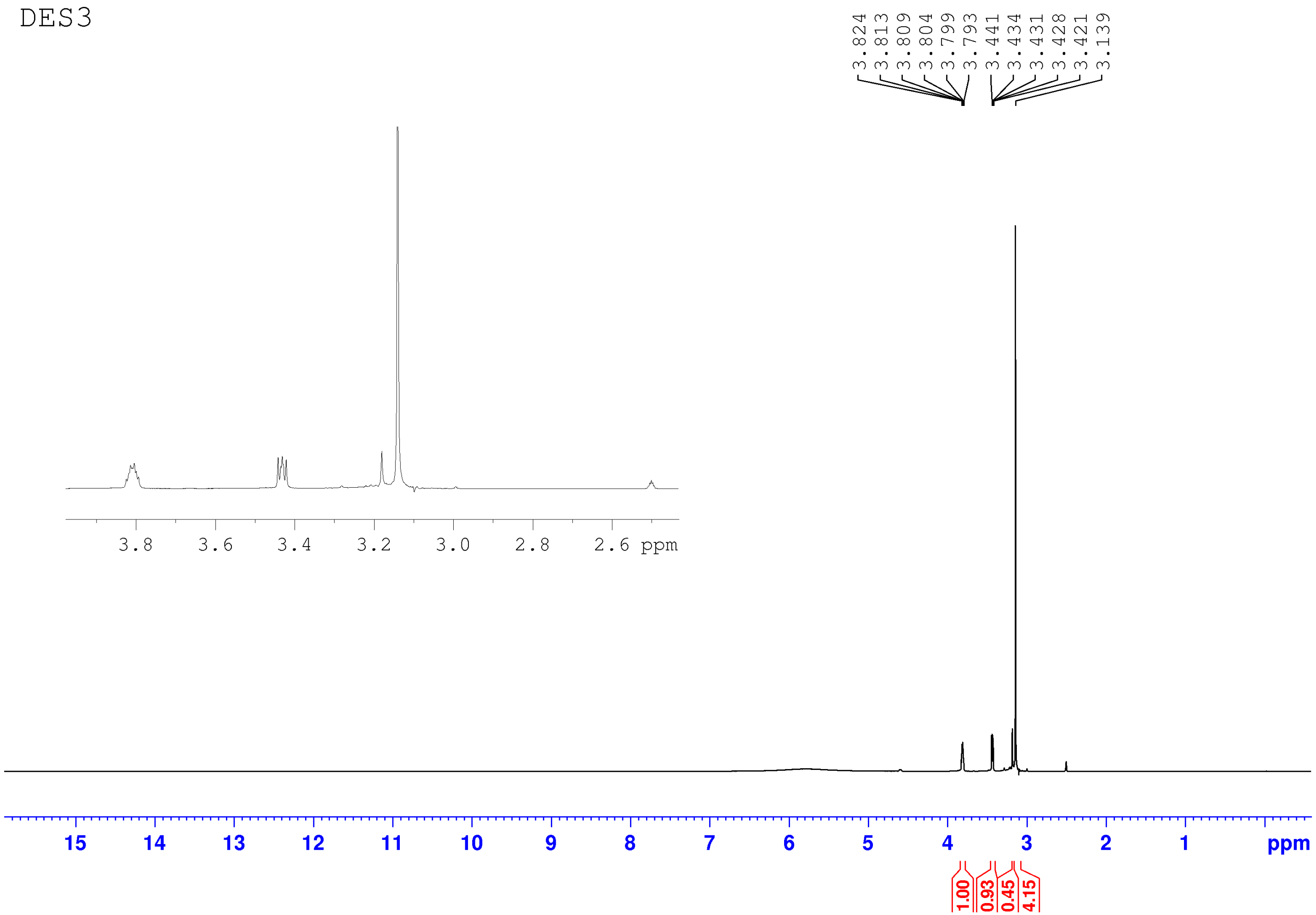

Figure S15. *¹H-NMR spectrum of DES3.*

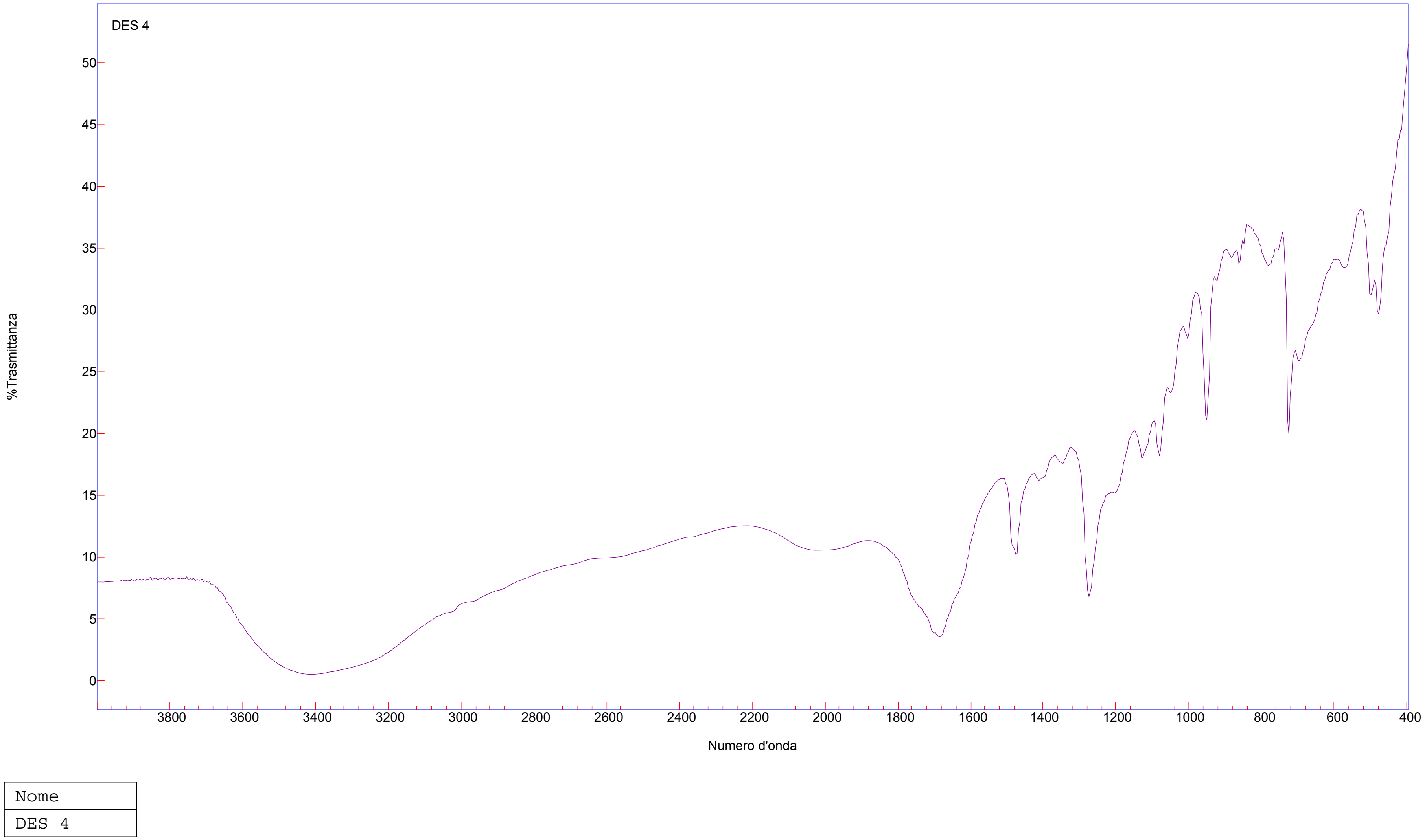

Figure S16. *FT-IR spectrum of DES4.*

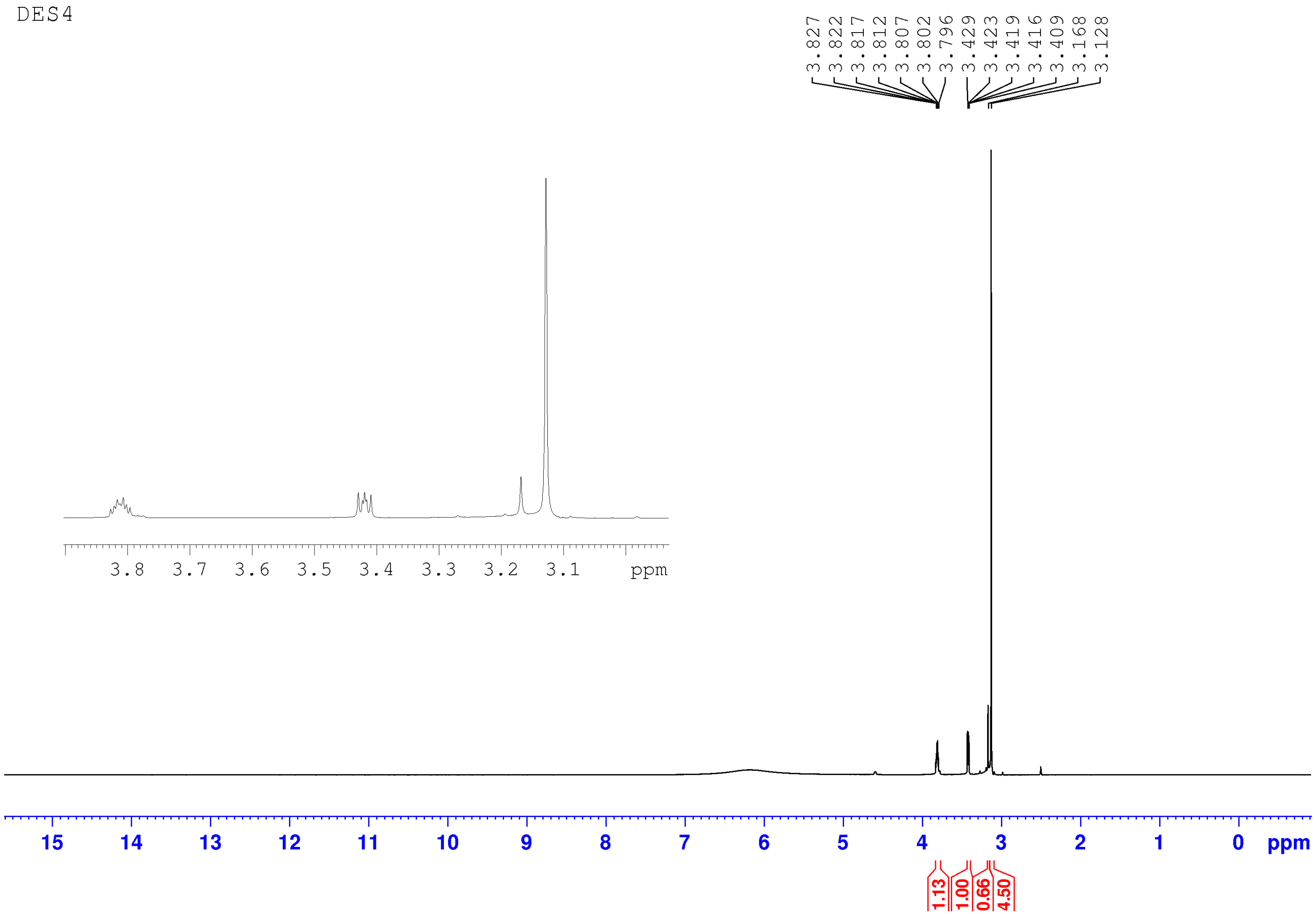

Figure S17. *^{1}H-NMR spectrum of DES4.*

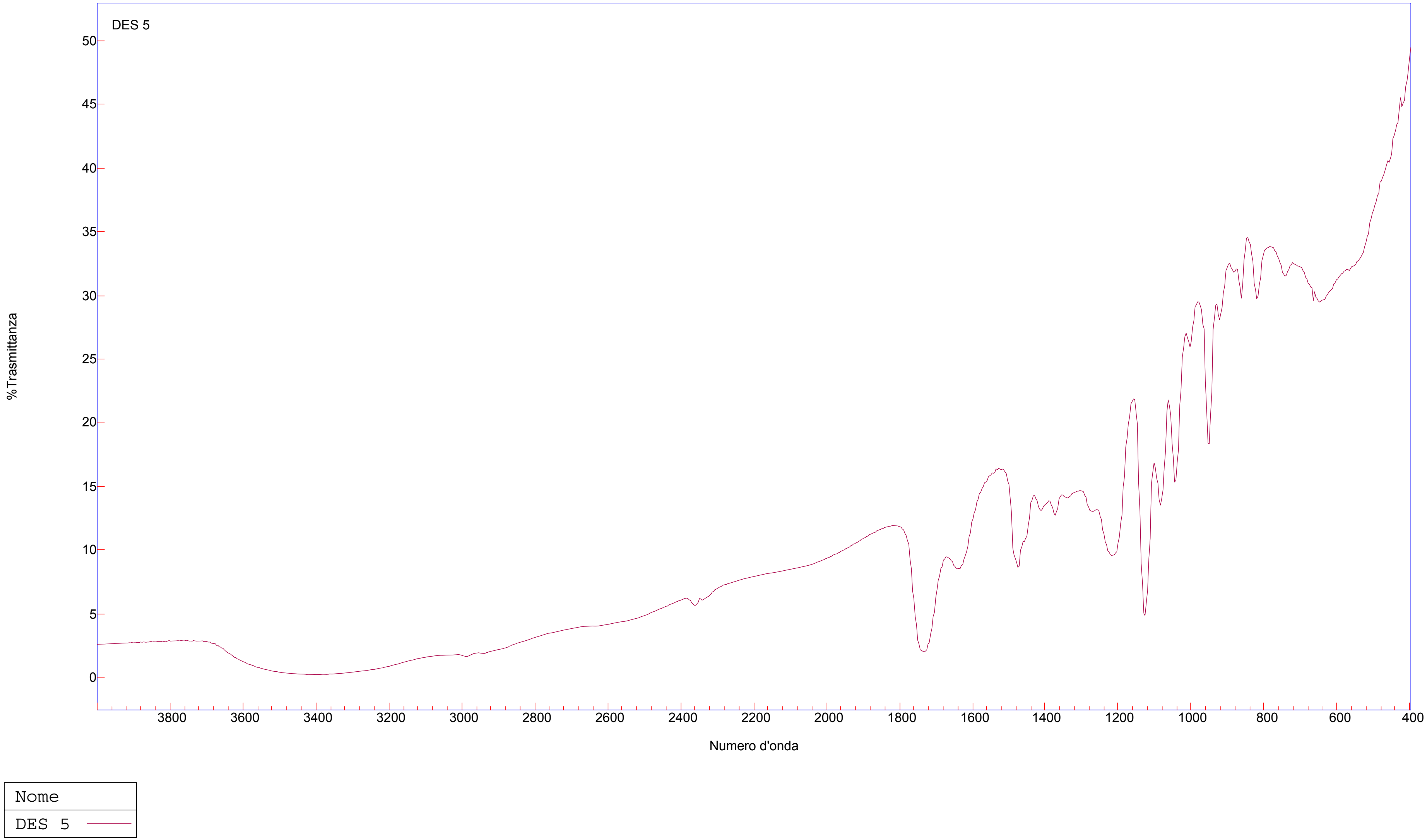

Figure S18. *FT-IR spectrum of DES5.*

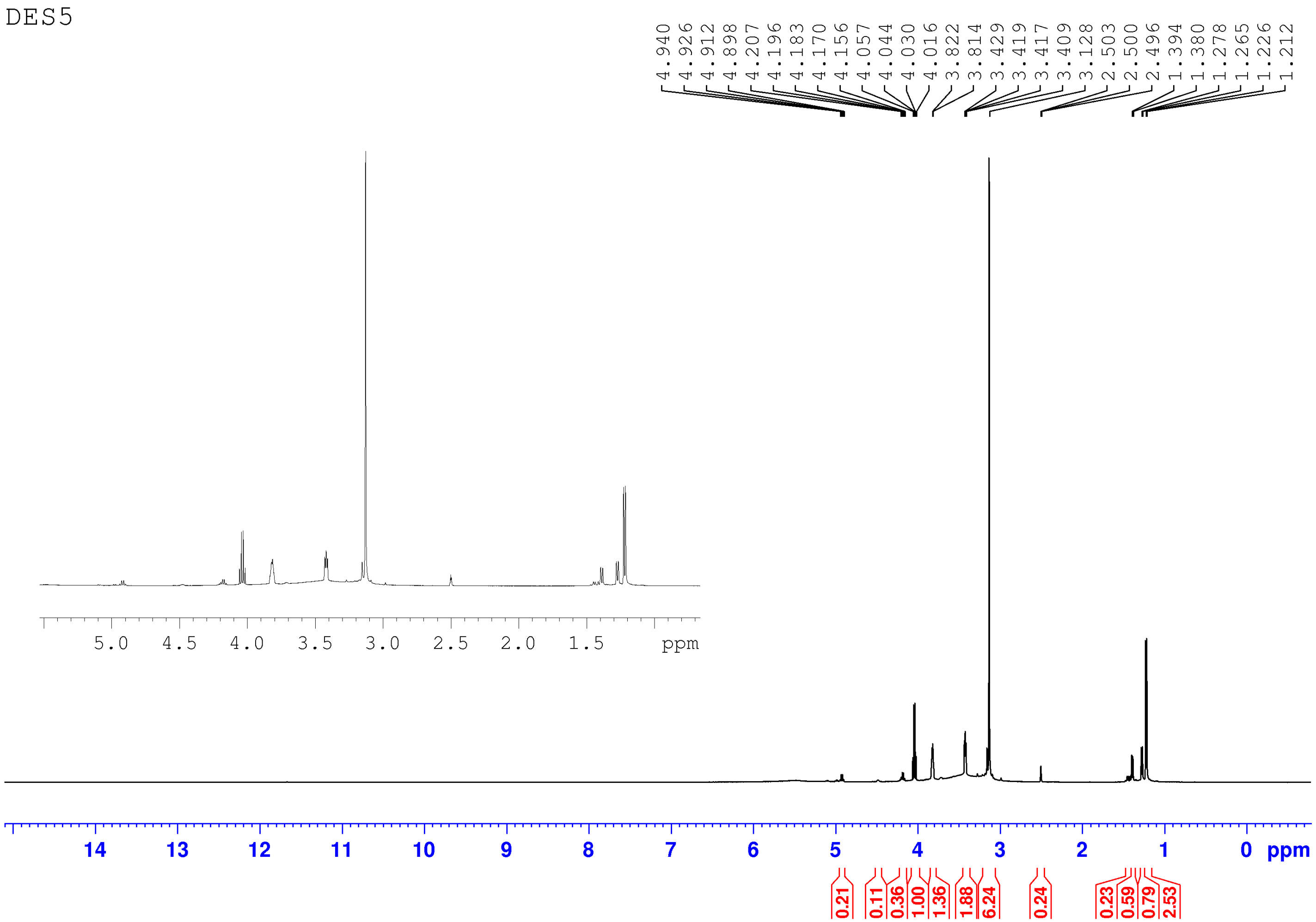

Figure S19. *^{1}H-NMR spectrum of DES5.*
